# Bupropion for the treatment of apathy in Huntington’s disease: A multicenter, randomised, double-blind, placebo-controlled, prospective crossover trial

**DOI:** 10.1371/journal.pone.0173872

**Published:** 2017-03-21

**Authors:** Harald Gelderblom, Torsten Wüstenberg, Tim McLean, Lisanne Mütze, Wilhelm Fischer, Carsten Saft, Rainer Hoffmann, Sigurd Süssmuth, Peter Schlattmann, Erik van Duijn, Bernhard Landwehrmeyer, Josef Priller

**Affiliations:** 1 Department of Neuropsychiatry, Charité-Universitätsmedizin Berlin, Berlin, Germany; 2 Department of Psychiatry, Charité-Universitätsmedizin Berlin, Berlin, Germany; 3 European Huntington’s Disease Network, Ulm, Germany; 4 Department of Neurology, University of Ulm, Ulm, Germany; 5 Department of Neurology, Huntington-Center NRW, St. Josef Hospital, Ruhr-University, Bochum, Germany; 6 Institute of Medical Statistics, Computer Sciences and Documentation, Jena Universityhospital, Jena, Germany; 7 Department of Psychiatry, Leiden University Medical Centre, Leiden, The Netherlands; 8 Cluster of Excellence NeuroCure, DZNE and BIH, Berlin, Germany; University of Toronto, CANADA

## Abstract

**Objective:**

To evaluate the efficacy and safety of bupropion in the treatment of apathy in Huntington’s disease (HD).

**Methods:**

In this phase 2b multicentre, double-blind, placebo-controlled crossover trial, individuals with HD and clinical signs of apathy according to the Structured Clinical Interview for Apathy—Dementia (SCIA-D), but not depression (n = 40) were randomized to receive either bupropion 150/300mg or placebo daily for 10 weeks. The primary outcome parameter was a significant change of the Apathy Evaluation Scale (AES) score after ten weeks of treatment as judged by an informant (AES-I) living in close proximity with the study participant. The secondary outcome parameters included changes of 1. AES scores determined by the patient (AES-S) or the clinical investigator (AES-C), 2. psychiatric symptoms (NPI, HADS-SIS, UHDRS-Behavior), 3. cognitive performance (SDMT, Stroop, VFT, MMSE), 4. motor symptoms (UHDRS-Motor), 5. activities of daily function (TFC, UHDRS-Function), and 6. caregiver distress (NPI-D). In addition, we investigated the effect of bupropion on brain structure as well as brain responses and functional connectivity during reward processing in a gambling task using magnetic resonance imaging (MRI).

**Results:**

At baseline, there were no significant treatment group differences in the clinical primary and secondary outcome parameters. At endpoint, there was no statistically significant difference between treatment groups for all clinical primary and secondary outcome variables. Study participation, irrespective of the intervention, lessened symptoms of apathy according to the informant and the clinical investigator.

**Conclusion:**

Bupropion does not alleviate apathy in HD. However, study participation/placebo effects were observed, which document the need for carefully controlled trials when investigating therapeutic interventions for the neuropsychiatric symptoms of HD.

**Trial registration:**

ClinicalTrials.gov 01914965

## Introduction

Apathy is a common behavioral syndrome in neuropsychiatric disorders with prefrontal cortex (PFC) and basal ganglia (BG) pathology, such as Huntington’s disease (HD) [1, 2]. It is broadly defined as the primary absence of motivation, lack of initiative and drive, as well as emotional indifference [3]. Apathy can be divided into three major syndrome domains—deficient emotional-affective function, cognitive function, or auto-activation [2].

In HD, apathy is the most common neuropsychiatric syndrome that correlates directly with disease progression [4–6]. Loss of dopamine (DA) receptor expression in fronto-striatal circuits was proposed as a key pathophysiological mechanism of apathy in HD [7, 8]. Neurodegeneration begins in the striatum as early as 15 years prior to motor onset, and then extends to frontal and PFC cortex regions [9–11]. Pathological changes in the orbital and medial PFC and the projections to limbic brain regions, predominantly the ventral striatum (VS), have been associated with the development of apathy in HD [2]. Magnetic resonance imaging (MRI) is capable of measuring atrophy [12–14] as well as alterations in mesolimbic DA processes [15, 16], which are linked to anticipation and processing of reward or punishment. In premanifest HD patients, an aberrant ventral striatal response during a monetary incentive delay task has been observed [17].

Despite of the high prevalence and disease burden of apathy in HD, research on therapeutic options for apathy is rare, and no effective treatment is at hand [18, 19]. This is the first controlled trial (CT) on the treatment of apathy in HD. It was the aim of this trial to evaluate the efficacy and safety of bupropion in the treatment of apathy in HD. We chose the antidepressant bupropion for its mode of action of blocking norepinephrine and DA reuptake, thereby potentially increasing DA neurotransmission in areas relevant for apathy. In addition, several single case reports and results of small series suggested the effectiveness of bupropion for the treatment of apathy in HD and other neurodegenerative diseases [20–22]. In addition, we investigated the effect of bupropion on DA-associated reward processing in an established gambling task using fMRI [23, 24].

## Materials and methods

ACTION-HD (Apathy cure through Bupropion in Huntington’s disease) is a multi-center, randomized, double-blind, placebo-controlled, 2x2 crossover phase 2b investigator-initiated trial (IIT) that was conducted at four sites in Germany between May 2012 (recruitment of first patient) and May 2014 (last patient leaving the trial). The ACTION-HD trial was registered at the EudraCT clinical trial register (EudraCT number 2009-013698-16) on 24^th^ March 2011 prior to inclusion of the first patient. We later registered the trial at clinicaltrials.gov. The authors confirm that all ongoing and related trials for this drug/intervention are registered. The protocol for this trial is available as supporting information; see S1 Clinical trial protocol.

### Ethics statement

The study was registered and approved by the German Competent Authorities (Bundesinstitut für Arzneimittel und Medizinprodukte (registration number 61-3910-4037522; 16.01.2012) and the Ethics Commission of the State of Berlin (Ethik-Kommission des Landes Berlin, Landesamt für Gesundheit und Soziales; registration number 11/0351- ZS EK; 27.01.2012), Berlin, Germany, as well as the institutional review boards of the Universities of Bochum, Münster and Ulm (Clinical Trial protocol version 1.1. [17.11.2012], version 2.0 [amendment 2; 22.02.13]; patient informed consent form version 2.0 [17.11.2011], version 3.0 [amendment 2; 22.02.13]; informant informed consent form version 1.0 [17.11.2011]). The study was conducted in accordance with the ethical principles laid out in the Declaration of Helsinki (1996) and consistent with Good Clinical Practice. All study participants provided informed consent before any study-related procedures were undertaken.

### Study participants

After signing the informed consent form, genetically verified HD patients aged 25 to 75 years and informants were included in the trial. Apathy was ascertained by the Structured Clinical Interview for Apathy—Dementia (SCIA-D). A caregiver living in close proximity to the patient had to participate as informant. Patients with clinically significant depression (NPI depression score >4), schizophreniform psychosis, a Mini-Mental State Examination (MMSE) < 18, marked chorea bucco-oro-lingual, of face, trunk or extremities, active suicidality based on the answer “yes” to questions 4 and 5 of the “suicidal ideation” section of the Columbia-Suicide Severity Rating Scale (C-SSRS), or treatment with antipsychotics other than tiapride, MAO-B inhibitors, amantadine, levodopa, D- or D,L-amphetamine or psychostimulants within 1 month prior to the first dose were excluded. A full list of inclusion and exclusion criteria is provided in S1 Clinical trial protocol in the supporting information. The allocation to either of two treatment arms was based on a central randomization code generated by means of the randomization procedure of nQuery 7.0. and performed by the study biometrician (PS).

### Primary and secondary objectives and sample size justification

The primary objective was to determine the influence of bupropion compared to placebo on the change of apathy as quantified by the informant-based Apathy Evaluation Scale (AES)-I, where I [informant] is a family member or friend familiar with the daily activities of the subject (score: 18–72 points; adjusted for the baseline values at week 0 or 13, respectively). Due to lack of published data, the power calculation was based on an unpublished patient cohort (n = 50) followed at the Centre for Brain Repair, University of Cambridge, UK (data generously provided by Sarah L. Mason and Roger A. Barker) for three years, where the AES-I showed a mean score of 31 points (standard deviation SD = 15.6). We defined clinically significant improvement as a 35% reduction of the mean AES-I score and calculated an absolute effect size of 0.35*31 points = 10.85 points. An estimate of 10 points was used for sample size determination and a within subjects SD of 15.0 was assumed. Accordingly, with a sample size in each group of 19 and a 2x2 crossover design, we would have 80% power to detect a difference in means of 10.00 (the difference between a Treatment 1 mean, μ1, of 31 and a Treatment 2 mean, μ2, of 21) assuming that the crossover ANOVA √MSE is 15.00 (the standard deviation of differences is 21.21) using a two group t-test (Crossover ANOVA) with a 0.05 two-sided significance level. In order to account for potential dropouts, we decided to randomize 40 patients. Sample size calculation was performed with nQuery 7.0.

The secondary objectives included an assessment of: 1. the safety and tolerability of bupropion in HD, 2. the influence of bupropion compared to placebo on the change of apathy as quantified by the AES-C (clinician) or the AES-S (self), and the NPI and UHDRS apathy scores, 3. the change of neuropsychiatric symptoms (UHDRS, HADS-SIS), 4. the change of cognitive performance (UHDRS and MMSE), 5. the total motor score (UHDRS), 6. the change of activities of daily living (UHDRS), 7. the change of the NPI caregivers’ distress score (NPI-D), and 8. the change of VS and ventromedial PFC activation in response to a gambling task as quantified by fMRI.

### Dosing and study plan

Study participants and informants took part in 8 study visits S1 Fig. Prior to and after cross-over (week 11), all study participants received once daily 150 mg bupropion or placebo for 2 weeks, followed by 300 mg bupropion or placebo per day for 8 weeks. In the case of intolerable side effects after the dose increase to 300 mg bupropion or placebo, dosing was reduced to 150 mg and maintained for the duration of the treatment arm.

### Assessments

Assessment sequence: Apathetic HD patients are likely to have progressed disease [5], restricted cognitive resources, a short attention span and may experience fatigue during study-related procedures. We therefore designed a hierarchy of assessments that took these potential limitations into account.

Assessment of demographic characteristics including medical history, concurrent medication and physical examination was performed in a structured fashion based on the EHDN platform, which was approved by the institutional review boards of the participant centers for the observational REGISTRY study of the EHDN.

#### Structured Clinical Interview for Apathy—Dementia (SCIA-D)

To diagnose apathy and rate the severity of apathy, the SCIA-D was performed at screening, prior to and after each treatment interval. The SCIA-D was originally validated in patients with AD. It assesses seven domains relative to the individual’s previous level of functioning and incorporates the judgment of the informant [3, 25]. A German version (Translation: S. Forstmeier, M. Mortby, 2008) authorized by S.E. Starkstein (University of Western Australia, Australia) was used.

#### Apathy Evaluation Scale (AES)

To rate symptoms of apathy, the AES was filled in by the informant (AES-I; primary outcome variable), the study participant (AES-S), and the clinical investigator (AES-C) after a semi-structured clinical interview of participant and informant by the clinical investigator at the beginning of each study visit. Participant and informant had to perform the AES separately. Both were instructed not to discuss the answers. The AES scale consists of 18 items, each of which is assessed on a four-point Likert scale, so that the total score ranges from 0 to 54 [26, 27]. The AES was not used as an inclusion criterion, as there is no validated cut-off for the presence of clinically relevant apathy in HD. To ensure uniformity of SCIA-D and AES procedures, we produced a teaching video. In addition, study personnel were trained in published guidelines for performance [27]. To minimize inter-rater variability, virtually every study participant was rated by the same clinical investigator with longstanding experience in HD during the entire course of the study.

#### Neuropsychiatric Inventory (NPI)

To exclude comorbid depression, a “depression/dysphoria” score of ≤4 on the NPI was required for study inclusion (maximum score is 12 [frequency x severity]). To evaluate effects and side effects of the study medication, the domains “depression/dysphoria”, “apathy/indifference” and “irritability/lability” were assessed prior to and after each treatment episode. In addition, caregiver distress (NPI-D) was assessed from 0 (not at all) to 5 (extremely).

#### The Hospital Anxiety and Depression Scale combined with the Snaith Irritability Scale (HADS-SIS)

To test for symptoms of depression and signs of irritability, the HADS-SIS was performed prior to and after each treatment episode. The HADS is a self-report questionnaire that consists of 14 items rating symptoms of depression (7 items) and anxiety (7 items). The SIS rates inward and outward irritability (8 items). Each item is rated on a four-point scale.

#### Unified Huntington’s Disease Rating Scale (UHDRS)-99

The UHDRS-99 was performed prior to and after each treatment episode. The UHDRS-99 is an extension and update of the UHDRS, the most commonly used and validated clinical rating scale to assess motor (including chorea and dystonia), psychiatric, and cognitive (Stroop, Verbal Fluency Test, Symbol Digit Modalities Test) symptoms of HD, and to determine functional capacity [28].

#### Columbia-Suicide Severity Rating Scale (C-SSRS)

To exclude suicidal ideation or behavior, this semi-structured scale was performed during every study visit and telephone interview.

#### Mini Mental Status Examination (MMSE)

To assess the cognitive state of the study participant, the MMSE was administered prior to and after every treatment episode.

### Structural and functional magnetic resonance imaging

#### Gambling task

To assess brain responses and functional connectivity during reward processing by fMRI, an established gambling task known to elicit strong dopaminergic responses in the VS was used [24, 29]. A detailed description of MRI parameters, determination of regions of interest (ROI), gambling task paradigm and the applied image-processing pipeline is provided in the S1 Supplemental methods and S2–S4 Figs.

### Data collection and statistical analysis

Data collected on each patient were recorded on an electronic case report form (eCRF) based on the EHDN REGISTRY platform. During the study, a 100% monitoring was performed in all centres and eCRF data entries were checked using computerized and manual means to identify problem fields. All study-related documentation including the study master file was collected in an ACTION-HD study section of the EHDN web portal accessible to all study personnel. During the entire study, weekly telephone conferences were held by the study coordination team.

#### Statistical analysis

For efficacy analysis, the confirmatory inferential statistical evaluation of the primary target parameter was based on a linear mixed effects model with the AES-I score as dependent variable. The model used treatment group and period as fixed factors, and the baseline AES-I score as covariate. Random effects were introduced for the intercepts, thus taking the correlation within participants into account. All secondary efficacy variables were primarily evaluated by using descriptive statistics. If applicable, a similar analysis was performed for secondary variables in an explanatory manner. A detailed description of image analysis procedures is provided in the S1 Supplemental methods. An excerpt of the data that will allow full analysis of all results shown in the manuscript except for patient-relevant data that are protected by confidentiality regulations are available to all interested researchers by contacting the EHDN Scientific and Bioethics Advisory Committee (SBAC) at actionhd_data-request@euro-hd.net.

#### Safety analysis

The safety analysis was performed as outlined in the study protocol (S1 Clinical trial protocol, page 23–30). After the last patient left the study and prior to the database closure, protocol deviations were assessed by the site investigators during a blind data review meeting in April 2014 and classified as “minor” or “major” based on joint decisions.

## Results

**Fig 1:**
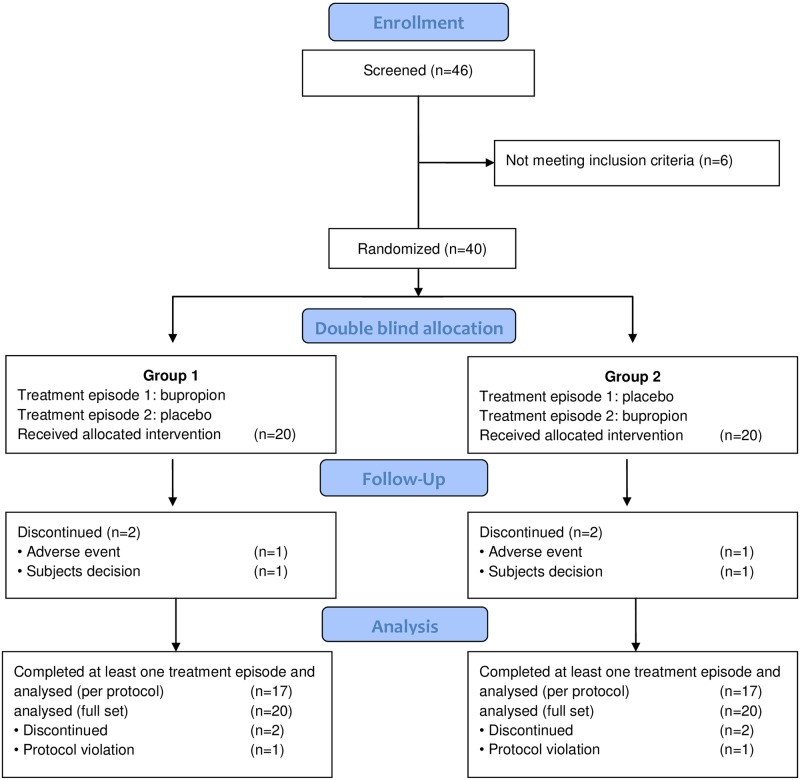
CONSORT flow diagram.

Between May 2012 and October 2013, 40 apathetic HD patients were randomized into two treatment groups (20/20) Fig 1. The subject characteristics are summarized in Table 1. Eighteen study participants consented to participate in the adjunct MRI study. The last study participant finished the study in April 2014. The study database was locked on May 30, 2014. Other than duration of disease (group 1: 8.4 ± 3.6 years vs. group 2: 5.5 ± 2.4 years; p<0.05), there were no statistically significant treatment group differences for any demographic parameter or baseline values of primary or secondary outcome variables Table 1.

**Table 1:** Demographic data and neuropsychiatric profiles (full analysis set).

	Group 1 (bupropion/placebo)^b^	Group 2 (placebo/bupropion)^b^	
Number of participants^a^	20	20	
Age, years (mean ± SD)^c^	54.6 ± 8.5	54.3 ± 10.7	
Males (number)	11	15	
Weight, kg (mean ± SD)	78.4 ± 14.4	80.7 ± 18.7	
BMI (mean ± SD)	25.1 ± 3.2	26.5 ± 5.2	
CAG repeats (mean ± SD)	43.6 ± 2.3	43.9 ± 3.7	
Duration of disease (years)	8.4 ± 3.6	5.5 ± 2.4^d^	p<0.05
Concomitant medication with tiapride	11	11	
UHDRS-Motor (mean ± SD)	39.3 ± 12.3	34.1 ± 12.6^e^	
TFC (mean ± SD)	7.40 ± 2.7	8.8 ± 3.5	
**Apathy**			
AES-I (mean ± SD)	36.4 ± 7.9	34.2 ± 7.7	
AES-S (mean ± SD)	29.3 ± 4	22.7 ± 8.6^e^^,^ *	
AES-C (mean ± SD)	36.0 ± 6.9	32.6 ± 7.9^e^^,^ *	
NPI-Apathy (mean ± SD)	8.8 ± 2.1	8.7 ± 2.1	
UHDRS-Apathy (mean ± SD)	10 ± 2	9.4 ± 3	
**Depression/irritability**			
NPI-Depression (mean ± SD)	0.7 ± 1.1	0.8 ± 1	
HADS-Depression (mean ± SD)	9.7 ± 4.6^e^	8.2 ± 4.1	
NPI-Irritability (mean ± SD)	1.5 ± 1.9	2.2 ± 2.1	
SIS (mean ± SD)	5.1 ± 3.1^e^	6.1 ± 4.1	
**Cognition**			
Stroop interference (mean ± SD)	22.1 ± 9	20.5 ± 10.6	
SDMT (mean ± SD)	19.5 ± 8.6	22.1 ± 12	
VFT (mean ± SD)	17.3 ± 12.2	22.3 ± 14.0	
MMSE (mean ± SD)	26.9 ± 2.7	26.4 ± 3	
**Distress caregiver**			
NPI-D (apathy) (mean ± SD)	3.3 ± 1.0	3.5 ± 1.0	

* AES-S vs. AES-I: t (df = 38) = -6.0504, p< 0.001, AES-S vs. AES-C: t (df = 38) = -5.6101, p-value = <0.001

^a^N includes all randomized patients

^b^Patients were allocated to group1 (1st treatment episode: bupropion, followed by washout and crossover, followed by 2nd treatment episode: placebo) or group 2 (1st treatment episode: placebo, followed by washout and crossover, followed by 2nd treatment episode: bupropion).

^c^Values are given as mean ± SD (standard deviation)

^d^n = 18 (2 missing values)

^e^n = 19 (1 missing value)

Abbreviations: BMI = Body mass index; UHDRS = Unified Huntington’s Disease Rating Scale; TFC = Total Functional Capacity; AES-I = Apathy Evaluation Scale-informant (a friend or family member familiar with the daily activities of the subject); AES-S = AES-self; AES-C = AES-clinician; NPI = Neuropsychiatric Inventory; HADS = Hospital Anxiety and Depression Scale; SIS = Snaith Irritability Scale; SDMT = Symbol Digit Modalities Test; VFT = Verbal Fluency Test; MMSE = Mini Mental State Examination

A first important observation of the study was that the study participants rated their symptoms of apathy (AES-S) as significantly less severe than the caregivers (AES-I; t (df = 38) = -6.0504, p<0.001) or the clinical investigators (AES-C; t (df = 38) = -5.6101, p<0.001) Table 1.

Due to major protocol violations, two study participants had to be excluded from the per protocol analysis, because one did not follow the washout and crossover medication sequence between visit 4 and visit 4a, and the other took less than 80% of the study medication during the treatment period. Four participants discontinued the study; two for unwillingness to resume study visits, and one because of increased irritability while on bupropion. A fourth participant discontinued the study due to mood changes (cf. severe adverse events (SAEs), participant five).

As for the safety and tolerability of bupropion, 21 of 40 study participants had at least one of 56 AEs. Five AEs were rated as severe and 11 AEs were rated as moderate. The five SAEs resulted in hospitalizations: two after a fall, in one case with a shoulder fracture and an ensuing depression, a third for an elective varicose vein stripping, all on placebo. The fourth participant with a SAE was hospitalized after a short episode of lack of responsiveness three days after the ending of bupropion treatment (visit 4; S1 Fig). A fifth participant was hospitalized due to an increased instability of mood, irritability and aggression which evolved one day prior to the tapering of bupropion and while on concomitant medication with venlafaxine. The 11 moderate AEs included a mild to moderate increase in chorea and gait disorder (7 on bupropion/4 on placebo), an increase in irritability (1 on bupropion/6 on placebo), concentration difficulties (3 on placebo), a sleeping disorder (2 on bupropion), agitation and restlessness (1 on bupropion/1 on placebo), and an increase of perseverative thinking (1 on placebo). One study participant developed disinhibition, another hyperphagia with an increase of 20 kg of body weight while on bupropion. No participant developed active suicidality (answer “yes” to questions 4 and 5 of the “suicidal ideation” section of the C-SSRS), or indicated suicidal ideation upon questioning by the clinical investigators.

### Bupropion does not change apathy in HD as quantified by the AES-I

Comparing the primary outcome variable between the treatment groups using the linear mixed model adjusting for baseline, there was a slight but statistically non-significant change of the AES-I score due to bupropion treatment, which amounted to -0.81 (95% CI = -3.12, 1.52) in the full analysis set, and to -0.09 (95% CI = -2.56, 2.39) in the per protocol set Table 2.

**Table 2:** No change of apathy, irritability, cognition or function after 10 weeks of treatment with bupropion.

	Full analysis set		Per protocol set	
	Treatment effect [CI]	p-value	Treatment effect [CI]	p-value
**Apathy**				
**AES-I**^a^	**-0.81 [-3.1, 1.5]**	**0.5**	**-0.09 [-2.6, 2.4]**	**0.9**
AES-S	-2.15 [-4.6, 0.3]	0.1	-2.23 [-4.9, 0.4]	0.1
AES-C	-1.64 [-3.9, 0.7]	0.2	-1.26 [-3.7, 1.2]	0.3
NPI-Apathy	-0.79 [-1.8, 0.2]	0.1	-0.53 [-1.6, 0.5]	0.3
UHDRS-Apathy	-0.88 [-2.2, 0.4]	0.2	-0.71 [-2, 0.6]	0.3
**Depression/irritability**				
NPI-Depression	-0.62 [-1.3, 0.04]	0.07	-0.65 [-1.4, 0.1]	0.1
HADS-Depression	0.09 [-1.3, 1.5]	0.9	-0.16 [-1.7, 1.4]	0.8
NPI-Irritability	0.01 [-0.7, 0.8]	1	0.00 [-0.8, 0.8]	1.0
SIS (irritability)	-0.07 [-1.1, 1]	0.9	-0.20 [-1.3, 0.9]	0.7
**Cognition**				
Stroop (interference)	-0.03 [-1.9, 1.7]	1	0.38 [-1.3, 2]	0.6
SDMT	1.30 [-0.1, 2.7]	0.1	1.26 [-0.1, 2.6]	0.1
VFT	-0.16 [-2.1, 1.8]	0.9	-0.41 [-2.4, 1.6]	0.7
MMSE	0.59 [-0.2, 1.4]	0.1	0.77 [-0.1, 1.6]	0.1
**Distress caregiver**				
NPI-D (apathy)	0.14 [-0.3, 0.6]	0.5	0.21 [-0.2, 0.7]	0.3
**Functional scores**				
UHDRS-Motor	-0.64 [-2.7, 1.5]	0.5	-0.45 [-2.7, 1.8]	0.7
TFC	0.33 [-0.1, 0.7]	0.1	0.38 [0, 0.8]	0.07
UHDRS-Function	0.29 [-0.3, 0.9]	0.3	0.29 [-0.3, 0.9]	0.4
UHDRS-Independence	-0.53 [-2.2, 1.1]	0.5	-0.59 [-2.3, 1.2]	0.5

^a^Results are given as treatment effect and 95% confidence interval. **The primary outcome parameter is shown in bold**. Inferential statistical evaluation of the primary and secondary outcome parameters were based on a linear mixed effects model with the AES-I score as dependent variable using only the first baseline before treatment. The significance level was set to 0.05.

In any case, this level of change had no clinical relevance (defined as a change of 10 points or more on the AES, cf. Materials & methods). The results did not change when using multiple imputations to take missing values into account. Including random effects for the site produced a corresponding variance of random effects equal to zero, showing that there was no relevant heterogeneity between sites. When we grouped the AES questions according to the three major apathy syndrome domains (cognitive, behavioral, emotional) and analyzed them separately, we still failed to observe a significant treatment effect on any of the three apathy domains. However, when looking at each AES question separately, a significant treatment effect was observed for question 2 “he/she get things done during the day” (-0.22 [95% CI = -0.44, 0.01; p<0.05]).

### Bupropion does not change apathy in HD as quantified by the AES-S, AES-C, NPI-Apathy or UHDRS-Apathy

For the secondary outcome parameters used to quantify apathy in this study, we observed a slight treatment effect of bupropion measured by the AES-C (-1.26 [95% CI = -3.7, 1.2]), the NPI-Apathy (-0.53 [95% CI = -1.6, 0.5]) and the UHDRS-Apathy (-0.71 [95% CI = -2, 0.6]), none of which were statistically significant or clinically relevant Table 2. The study participants themselves rated a reduction of apathy in the AES-S (-2.23 [95% CI = -4.9, 0.4]), which was not significant or clinically relevant Table 2. When we grouped the AES-C and AES-S questions according to the three major apathy syndrome domains, we again did not observe a significant treatment effect on any of the three apathy domains. Looking at the questions separately, a significant bupropion treatment effect was revealed for question 17 “he/she has initiative” of the AES-C (- 0.25 [95% CI = -0.5, 0; p<0.05]).

### Bupropion does not influence motor performance, irritability, cognition, or function in HD

No significant changes in irritability (HADS-SIS, NPI-Irritability) or motor function (UHDRS-motor) due to treatment with bupropion were observed Table 2. This was somewhat surprising as some clinical investigators noted an increase in irritability and chorea in some study participants during the trial, as has been described in a case series. However, this could not be attributed to bupropion after unblinding of the trial. Cognition, in particular executive function, did not improve under medication with bupropion. Moreover, the UHDRS-Function assessment tool and the UHDRS-Independence scale did not reveal any significant changes in the activities of daily living as a result of bupropion treatment Table 2. Finally, caregiver distress was not alleviated by treatment of apathetic HD patients with bupropion Table 2. Taken together, there was no change of any clinical secondary outcome parameter as a result of bupropion treatment.

### Trial participation/placebo effects alleviate symptoms of apathy

Irrespective of the intervention (bupropion or placebo), informants and clinical investigators observed a significant improvement of the symptoms of apathy (AES-I, AES-C) or the apathy syndrome (NPI-Apathy, UHDRS-Apathy) predominantly during the first treatment period Table 3. Notably, this was not observed in the AES-S scores.

**Table 3:** Change of apathy after the first treatment period suggests activation through trial participation.

Instrument	Score (min/max) ^a^	Baseline^b^	Period 1^b^	Period 2^b^	p-value
AES-I	0–54	35.3 ± 7.8	32.6 ± 9.5	31.1 ± 10.3	< 0.005^c^
AES-S	0–54	26.1 ± 8.6	26.5 ± 8.3	26.3 ± 9.5	0.9
AES-C	0–54	34.4 ± 7.5	32 ± 8.4	31 ± 9.3	< 0.05
NPI-Apathy	1–12	8.6 ± 2.5	7 ± 3	6.7 ± 3.3	< 0.001
UHDRS-Apathy	0–16	9.7 ± 2.5	8.7 ± 3.17	7.9 ± 3.9	< 0.05
NPI-D Apathy	0–5	3.35 ± 1	3.1 ± 1.1	2.7 ± 1.2	< 0.01

^a^Minimum and maximum values of the instruments used to grade apathetic symptoms (AES), apathy as syndrome (NPI, UHDRS) or caregiver distress due to apathy (NPI-D)

^b^Values are given for the full analysis set as means ± SD (standard deviation)

^c^p = 0.14 after multiple imputation to take missing values into account

Study participants also rated their symptoms of apathy as significantly less severe than the caregivers or the clinical investigators Table 1. There was also a tendency of a period effect when grading irritability by the UHDRS-Behavior, the SIS or the NPI. However, the observations were not consistent between instruments, and the changes in irritability scores were too low to have any clinical relevance (data not shown).

### Bupropion does not influence brain structure, brain response or brain connectivity

The analyses of structural and functional MRI data revealed no significant treatment effects. We only found a trend toward a decline in the right orbitofrontal cortex gray matter for the bupropion group (ANOVA p = 0.088), and an increase in functional connectivity between the left and right ventral striatum for the bupropion group (ANOVA p = 0.068) S1, S2 and S3 Tables.

## Discussion

ACTION-HD is the first randomised controlled clinical trial on the treatment of apathy in HD. We did not find a clinically significant effect of a 10-week treatment course with bupropion (at a dose of 300 mg per day) on the severity of apathy in HD based on assessments by informant, study participant and clinical investigator using the AES. Two further informant-based assessment tools (NPI-Apathy and UHDRS-Apathy) also failed to reveal a treatment effect of bupropion.

A concern when designing this trial was the lack of robust longitudinal data on the severity of apathy in HD, since no ‘gold standard tool’ for the assessment of apathy exists in general [30], and for HD in particular. We chose the SCIA-D as a screening instrument for the diagnosis of apathy, and the AES as a baseline and follow-up tool to measure the severity of apathy. The SCIAD-D is based on the criteria of Starkstein et al. [25], which were recently incorporated into the consensus criteria for the diagnosis of apathy proposed by an international task force [31]. The criteria refer to the prior functional status of the study participant, which we judged to be more appropriate than an arbitrarily determined baseline cutoff score. The AES is the most widely used rating scale for the symptoms of apathy. Clinical studies have also relied on single item measures like the UHDRS-Behavior or the NPI. Only recently, the AES has been validated in HD [32]. Importantly, confounding factors like lack of awareness [33], comorbid depression, motor symptoms, or side effects of concomitant medication have to be considered when evaluating apathy in HD.

The reasons for choosing the AES as the primary rating scale in the ACTION-HD trial included i. the availability of a validated German version [34], ii. the possibility of separate ratings by participant, informant and clinician, iii. the good inter-rater reliability for the AES-C [35], iv. the separate evaluation of all three domains of apathy [2, 26], v. the provision of performance guidelines by the author, allowing for further standardization [27], vi. the suitability of the AES for statistical evaluation due to the 4-point Likert scale, vii. the reasonable length and complexity, allowing for informative answers by cognitively impaired study participants. Based on previous reliability studies in AD, the test-retest reliability is 0.94 for the AES-I, 0.88–0.89 for the AES-C, and 0.76 for the AES-S [27, 36]. Power calculations were performed using longitudinal AES data from a recently published HD cohort [32]. AES threshold scores have been suggested for AD with a range from ≥ 30 to ≥ 41.5 [37], but also for conditions like traumatic brain injury [38]. Baseline scores of some of our patients were below these values Table 1, but there was no doubt about the presence of apathy in these study participants based on the SCIA-D as well as on the clinical assessments. Moreover, we found a mean NPI apathy score of 8.6 at baseline in our study. NPI apathy scores above 4 have recently been suggested to define clinically significant apathy in dementia [39].

We chose the informant version of the AES, the AES-I score, as the primary outcome variable since we assumed that HD patients rate their symptoms of apathy less severe than their caregivers or physicians [40, 41]. In HD, like in other neuropsychiatric diseases with frontal pathology, lack of insight into symptoms and functional capacities is common [42, 43]. Marin originally evaluated the AES in several diseases, including AD [26, 27], where he observed that patients graded the symptoms of apathy as less severe than their caregivers or clinicians. An association with frontal dysfunction has been suggested also for AD [44]. On the other hand, one has to consider that caregivers may be biased by the burden that the patient’s apathy bestows on them [45]. As expected, participants of Action-HD rated their symptoms of apathy as significantly less severe than their caregivers or the clinical investigator. In addition, a significant period effect was observed for AES-I and AES-C but not for AES-S scores Tables 1 and 3. In contrast to these findings, Mason and Barker at the University of Cambridge (UK) did not find a significant difference between AES-I and AES-S sores during an observation period of 19 months on average [32]. An explanation for this discrepancy could be that we physically separated patients and informants when performing the AES, as we previously observed that patients may adapt their judgements to the ratings of the caregivers when assessed in the same setting. In addition, the relative number of participants with a TFC score between 7 and 10 was small in the Cambridge study, whereas the mean TFC scores in our study were 7.4 and 8.8 in the intervention groups, respectively. Of the 40 patients rated as apathetic by their informants, 21 were lost to follow up in the Cambridge study, which creates a bias. Moreover, we excluded patients with dopamine-depleting drugs, and patients with comorbid depression in our study in order to reduce confounding effects. This has also recently been recommended by Cummings and coworkers [39]. However, we do acknowledge that HD patients with both depression and apathy may constitute a subgroup that responds better to bupropion than HD patients with apathy alone.

To quantify the affective-emotional components of apathy we additionally used an established gambling task to measure reward perception as one of the major preconditions for motivation [24, 29, 46]. DA is thought to be involved in reward and motivation, possibly through modification of the mesolimbic and mesocortical pathways, as well as the orbitomedial PFC-striatal projections. The loss of DA receptor 1 and 2 expression in fronto-striatal circuits was proposed as a key pathophysiological mechanism of apathy in HD [7, 8]. Concurrent with the clinical results, we did not detect significant effects of bupropion treatment on brain responses and functional connectivity during reward processing in apathetic HD patients in our exploratory fMRI analysis. However, it is difficult to draw any conclusion from the small sample size.

When considering the study duration, we feel that 8 weeks on the full dose of bupropion should have been sufficient for the detection of a potential treatment effect. The duration of most randomized CTs on apathy in neurodegenerative diseases is between 6 and 12 weeks. In fact, Cummings et al. recommended a study duration of 8 to 12 weeks when designing clinical trials for apathy in neurodegenerative diseases since the worsening of apathy in both the verum and placebo arms may result in an underestimation of an initial treatment effect in the intervention arm if the intervention period would be extended beyond 12 weeks [39].

It is possible that bupropion did not reach sufficient concentrations within the central nervous system at a daily dose of 300mg. However, we felt that daily doses of 450 mg could be problematic in advanced stages of HD as the risk of seizures, suicidality and irritability may increase.

HD patients in more advanced disease stages often require symptomatic treatment [19]. We cannot exclude the possibility that concomitant medication in some of the study participants may have aggravated apathy or mitigated the effect of bupropion. This is particularly true for tiapride, a dopamine D2 and D3 receptor antagonist, which is commonly used to treat chorea in Germany. A type 2 error may also result from low sensitivity of the available instruments for grading apathy. In the case of the AES, the poor differentiation between the answers 2 (“slightly characteristic”) and 3 (“somewhat characteristic”) on the four point Likert scale may reduce sensitivity. It is noteworthy that the validation of the AES in HD is still incomplete [41]. Our trial adds important data for this process.

Many recent drug trials in HD, e.g. the MermaiHD study [47] and the deutetrabenazine trial [48], have detected significant placebo effects. Interestingly, Cubo et al. observed a significant placebo effect on behavior, but not on motor symptoms or cognition in a large multicenter drug trial in HD [49]. This effect persisted over 3 years and was prominent if depression was absent. In Action-HD, we observed an improvement of the primary outcome measure AES-I for all study participants predominantly prior to crossover. This was replicated for the clinician-rated AES-C score. Study participants were informed about the crossover design. Since we compared period 1 and 2 data with the original baseline, the results suggest, that study participation/placebo effects have a positive impact on the severity of apathy. (cf. Table 3). Notably, the study participants themselves did not rate any improvement on the AES-S. These important findings indicate that our study was not underpowered to detect significant changes of apathy using the AES and that, when studying apathy in HD, activation by study participation should be considered when interpreting trial results.

## Conclusion

The ACTION-HD trial suggests that bupropion does not influence the severity of apathy in non-depressed HD patients as quantified by the AES and other behavioral assessment tools. We observed a significant period effect irrespective of the intervention (bupropion or placebo). These findings highlight the general need for controlled symptomatic treatment trials in HD.

## Supporting information

S1 CONSORT Checklist (DOC)

S1 Clinical trial protocol (PDF)

S1 Supplemental methods (DOCX)

S1 Fig Study design. (PDF)

S2 Fig Experimental set up: Slot machine paradigm. The period of interest (gain anticipation) is displayed in blue.
(PDF)

S3 Fig MR-image analysis pipeline. *Abbreviations*: MRI—Magnetic resonance imaging; Ss—single subject; MNI—spatial reference space as defined by the brain template of the Montreal Neurological Institute; ANCOVA—Analysis of covariance, MPRAGE—Magnetization prepared rapid-acquisition gradient-echo image; BOLD—Blood oxygenation dependent; GE-EPI—Gradient-echo echo-planar image, VBM—Voxel-based morphometry, SPM—Statistical parametrical mapping, TPM—Tissue probability map, GA—Gain anticipation, nGA—no Gain anticipation, ROI—Region of Interest, ANCOVA—Analysis of Covariance, VS—Ventral striatum, ACC—Anterior cingulate cortex, MPFC—Medial prefrontal cortex, OFC—Orbitofrontal cortex.
(PDF)

S4 Fig *A-priori* computed literature-based, probabilistic Regions Of Interest (ROIs). Displayed are tri-orthogonal cuts through the ROI center. The outer borders (2SD) of the probabilistic ROIs are displayed in green; the outer borders of the anatomical structures/constraints taken from the AAL-atlas are displayed in red. ROI name and the used anatomical constraints are listed left hand, MNI-center coordinates and volume of the ROIs are listed in the right hand part. *Abbreviations*: AAL—Automatically anatomical labeling, MNI—Reference space according to the brain template provided by the Montreal Neurological Institute, Tra—Transversal, Sag—Sagittal, Cor—Coronal, VS—Ventral striatum, ACC—Anterior cingulate cortex, MPFC—Medial prefrontal cortex, OFC—Orbitofrontal cortex, L—Left, R—Right.
(PDF)

S1 Table Treatment associated effects on brain structure (local gray matter volume). Interaction between TREATMENT and TIME as revealed by voxel-wise ANCOVA (p < .05 uncorrected, covariates age & sex). Alpha-errors adjusted post-hoc for ROI-volume. Post-hoc comparison V(pre<post) > P(pre<post). Abbreviations: FEW—Family-wise error, V—Verum, P—Placebo, R—Right, L—Left.
(DOCX)

S2 Table Treatment associated effects on brain response (GA—nGA). Interaction between TREATMENT and TIME as revealed by voxel-wise ANCOVA (p < .05 uncorrected, covariates chorea severity). Alpha-errors adjusted *post-hoc* for ROI-volume. Post-hoc comparison V(pre<post) > P(pre<post). Abbreviations: FEW—Family-wise error, V—Verum, P—Placebo, R—Right, L—Left.
(DOCX)

S3 Table Treatment associated effects on functional brain connectivity (PPI: GA—nGA). Interaction between TREATMENT and TIME as revealed by voxel-wise ANCOVA (p < .05 uncorrected, covariates chorea severity) for left VS (upper part) and right VS (lower part) separately. Alpha-errors adjusted *post-hoc* for ROI-volume. Post-hoc comparison V(pre< post) > P(pre<post). Abbreviations: PPI—Psycho-physiological interaction, VS—Ventral striatum, FEW—Family-wise error, V—Verum, P—Placebo, R—Right, L—Left.
(DOCX)

## Abbreviations

AD: Alzheimer’s disease
AE: adverse event
AES-C: Apathy Evaluation Scale-Clinician
AES-I: Apathy Evaluation Scale-Informant
AES-S: Apathy Evaluation Scale-Self
BG: basal ganglia
C-SSRS: Columbia-Suicide Severity Rating Scale
CT: controlled trial
DA: dopamine
fMRI: functional magnetic resonance imaging
HADS-SIS: The Hospital Anxiety and Depression Scale combined with the Snaith Irritability Scale
HD: Huntington’s disease
IIT: investigator initiated trial
MMSE: Mini Mental Status Examination
NPI: Neuropsychiatric Inventory
NPI-D: NPI-distress
PFC: prefrontal cortex
ROIs: regions of interest
SCIA-D: Structured Clinical Interview for Apathy—Dementia
UHDRS: Unified Huntington’s Disease Rating Scale
VS: ventral striatum
