# Supplementary material for: Bupropion for the treatment of apathy in Huntington’s disease: A multicenter, randomised, double-blind, placebo-controlled, prospective crossover trial
Source: PLoS One. 2017 Mar 21;12(3):e0173872. doi: 10.1371/journal.pone.0173872 (PMC5360242; doi:10.1371/journal.pone.0173872)
Supplement: S1 Clinical trial protocol — (PDF) [file pone.0173872.s002.pdf]

CLINICAL TRIAL PROTOCOL

PROTOCOL NUMBER: HDSY001
EUDRACT NUMBER: 2009-013698-16

Action-HD

(Apathy cure through Bupropion in Huntington's disease)

A randomized, double-blind, placebo-controlled prospective crossover trial investigating the efficacy and safety of the treatment with Bupropion in patients with apathy in Huntington's disease

Sponsor: Charité - Universitätsmedizin Berlin

Coordinating Investigator (National Principal Investigator):
Prof. Dr. Josef Priller
Abteilung für Neuropsychiatrie
Klinik für Psychiatrie und Psychotherapie
Campus Charité Mitte
Charité- Universitätsmedizin Berlin
Chariteplatz 1, 10117 Berlin, Germany
Phone: +49 30 450 617 209
Fax: +49 30 450 517 957
Email: josef.priller@chaite.de

Project Coordinator:
Dr. Harald Gelderblom
Abteilung für Neuropsychiatrie
Klinik für Psychiatrie und Psychotherapie
Campus Charité Mitte
Charité- Universitätsmedizin Berlin
Chariteplatz 1, 10117 Berlin, Germany
Phone: +49 30 450 617 239
Fax: +49 30 450 517 957
Email: harald.gelderblom@charite.de

Development Phase: II

Version/Date 1.1 / 17.11.2011

Confidentiality

The information provided in this document is property of Charité - Universitätsmedizin Berlin. It is strictly confidential and is available for review to investigators, potential investigators and appropriate Ethics Committees or Investigational Review Boards, Competent Authorities and Monitors. No disclosure should take place without written authorization.

Protocol Approval

The undersigned has read, reviewed and understood this protocol, including the appendices, agree that it contains all necessary details for carrying out this trial as described therein and will adhere to the Ethical and Regulatory Considerations stated.

_______________________________________ _____________________

Prof. Dr. Josef Priller
Coordinating Investigator/

Date

Declaration of the Principal Investigator

I have read this study protocol and agree that it contains all the information required for study performance. I agree to conduct the study as set out in the protocol. In particular, I agree to adhere to the moral, ethical and scientific principles of Good Clinical Practice, the Declaration of Helsinki in the version of 1996, the local laws and regulations and the applicable regulatory requirements.

I understand that all documentation that has not been previously published will be kept in the strictest confidence. This documentation includes the study protocol, Investigator’s Brochure, case report forms, and other scientific data.

Principal Investigator of the local study site

Prof. Dr. Josef Priller
Abteilung für Neuropsychiatrie
Klinik für Psychiatrie und Psychotherapie
Campus Charité Mitte
Charité- Universitätsmedizin Berlin
Charitéplatz 1, 10117 Berlin, Germany

Place / Date Signature

TRIAL CONTACTS

**MONITORING, DATA MANAGEMENT**	Katrin Barth<br>EHDN, Central Coordination<br>Oberer Eselsberg 45/1<br>89081 Ulm, Germany	phone +49 731 500 63108<br>fax +49 731 500 63082<br>e-mail katrin.barth@uni-ulm.de
**PROJECT MANAGEMENT**	Tim McLean<br>EHDN, Central Coordination,<br>PO Box 13820, West Linton, EH45 6AD, UK	phone +44 7738 433 165<br>fax +44 1968 481 232<br>e-mail tim.mclean@euro-hd.net
**STATISTICIAN**	Prof. Dr. P. Schlattmann,<br>Institut für Medizinische Statistik, Informatik und Dokumentation<br>Bachstr. 18<br>07743 Jena, Germany	phone +49 3641 934130<br>fax +49 3641 933200<br>e-mail peter.schlattmann@mti.uni-jena.de
**PHARMACY**	Cornelia Eberhardt<br>Apotheke - Klinische Prüfpräparate<br>Campus Virchow Klinikum<br>Charité - Universitätsmedizin Berlin<br>Augustenburger Platz 1<br>13353 Berlin, Germany	phone +49 30 450 561 104<br>fax +49 30 450 7 561 104<br>e-mail cornelia.eberhardt@charite.de
**SAFETY CONTACT**	Dr. Wilhelm Fischer<br>Klinik für Neurologie<br>Universität Ulm<br>Oberer Eselsberg 45<br>89081 Ulm, Germany	phone +49 731-500-63000<br>fax +49 731-500-63002<br>e-mail wilhelm.fischer@uniklinik-ulm.de

TABLE OF CONTENTS

LIST OF ABBREVIATIONS 8

PROTOCOL SUMMARY 9

SCHEDULE OF PROCEDURES 12

SCHEDULE OF TREATMENT 13

1 INTRODUCTION AND RATIONALE 14
2 STUDY OBJECTIVE 16
2.1 Primary objective 16
2.2 Secondary objectives 16
2.3 Study Design 16

3 PATIENT SELECTION 17
3.1 Number of Patients 17
3.2 Inclusion Criteria 17
3.3 Exclusion Criteria 17
3.4 Subject Completion and Withdrawal 18
3.4.1 Definitions 18
3.4.2 Procedure for Handling Withdrawals 19
3.4.3 Removal of Subjects from Therapy or Assessment 19

4 STUDY MEDICATION AND ADMINISTRATION 21
4.1 Study Medication 21
4.2 Dosage, Administration 21
4.3 Method of Blinding 21
4.4 Manufacturing and Packaging 22
4.5 Labelling and Preparation 22
4.6 Storage 23
4.7 Drug Accountability 23
4.8 Assessment of TREATMENT Compliance 23
4.9 Safety Evaluations 23
4.9.1 Clinical Laboratory Tests 23
4.9.2 For Female Patients 23
4.9.3 Adverse Events (AE) of Bupropion 24
4.9.4 Concomitant Medication / Treatment 25
4.9.5 Treatment after Completion of the Study 25

5 ADVERSE EVENTS 26
5.1 Eliciting and Documenting Adverse Events 26
5.2 Assessment of Severity 26
5.3 Assessment of Causality 27
5.4 Serious Adverse Events 28
5.4.1 Definition of Serious Adverse Events 28
5.4.2 Reporting of Serious Adverse Events 29

5.4.3 Follow-up of Adverse Events 29
5.5 Overdosage 29
5.6 Pregnancy 29
5.7 Breaking the Study Blind 30

6 STUDY PROCEDURES 31
6.1 Assessments 31
6.1.1 Unified Huntington's Disease Rating Scale (UHDRS) 31
6.1.2 Quantitative grip force and speeded tapping motor assessment 31
6.1.3 SCIA-D: (Structured Clinical Interview for Apathy - Dementia) 31
6.1.4 Apathy Evaluation Scale (AES) 32
6.1.5 Neuropsychiatric Inventory (NPI) 32
6.1.6 Hospital Anxiety and Depression Scale combined with the Snaith Irritability Scale (HADS-SIS) 32
6.1.7 Columbia-Suicide Severity Rating Scale (C-SSRS) 32
6.1.8 Mini Mental Status Examination 33
6.1.9 Functional MRI 33
6.1.10 12-lead ECG 33
6.1.11 Vital Signs 34
6.1.12 Laboratory Methods 34
6.2 Study visits 35
6.2.1 Screening Procedures (Visit 1, week -3 +/- 1 week) 35
6.2.2 Visit 2 (Baseline visit; day 0) 36
6.2.3 Visit 3 (2 weeks +/- 2 days after baseline visit [visit 2]) 36
6.2.4 Phone interview 1 (1 week +/- 2 days after visit 3) 37
6.2.5 Visit 4 (8 weeks (+/- 1 week) after visit 3) 37
6.2.6 Visit 4a (2 weeks +/- 2 days after visit 4) 38
6.2.7 Visit 5 (2 weeks +/- 2 days after visit 4a) 39
6.2.8 Phone interview 2 (1 week +/- 2 days after visit 5) 39
6.2.9 Visit 6 (8 weeks (+/- 1 week) after visit 5) 40
6.2.10 Visit 7 (Follow-up 3 weeks +/- 1 week after visit 6) 40
6.3 Data Collection 41
6.4 Study Monitoring 41
6.5 Data management 42

7 STATISTICAL METHODS AND DETERMINATION OF SAMPLE SIZE 43
7.1 Sample size and power Calculations 43
7.2 Method of Randomization 44
7.3 Statistical Evaluations 44
7.3.1 General Plan 44
7.3.2 Analysis Sets 45
7.3.3 Background and demographic characteristics 45
7.3.4 Study medication 46
7.3.5 Concomitant and Pre-study Medications 46
7.3.6 Efficacy Analyses 46
7.3.6.1 Primary efficacy variable 46
7.3.6.2 Secondary efficacy variables 47
7.3.7 Safety Analyses 49
7.3.7.1 Adverse Events 49
7.3.7.2 Laboratory Data 49
7.3.7.3 Vital Signs and Physical Examination Data 50

7.4 Interim ANALYSIS 50
7.5 Final Study Report 50
8 ETHICS AND INSURANCE 51
8.1 Ethical Conduct of The Study 51
8.2 Investigator Obligations 51
8.3 Independent Ethics Committee and Competent Authority 51
8.4 Subject Information and Consent 52
8.5 INSURANCE 52
8.6 Risk-Benefit-Analysis 53
8.7 Discontinuation of the study 53
9 USE OF INFORMATION AND PUBLICATION 55
10 ARCHIVING OF DATA / RECORDS OF STUDY 56
11 AMENDMENTS TO THE PROTOCOL 57
12 REFERENCES 58

LIST OF ABBREVIATIONS

AD	Alzheimer's disease
AE	adverse event
AES	apathy evaluation scale
BOL	bucco-oral-ligual
BP	blood pressure
CIOMS	Council for International Organizations of Medical Sciences
CA	competent authority
eCRF	electronic case report form
CAG	human genome nucleotides: Cytosine, Adenine; Guanine
CRO	contract research organization
CTA	clinical trial application
ECG	electrocardiogram
EHDN	European Huntington's Disease Network
FDA	Food and Drug Administration
FUP	follow-up
GCP	Good Clinical Practice
fMRI	functional magnetic resonance immaging
GCP-V	GCP-Verordnung, GCP-Ordinance
GmbH	Gesellschaft mit beschränkter Haftung, Ltd, limited liability company
HADS	Hospital Anxiety and Depression Sacle
HD	Huntington's disease
β-HCG	β human chorionic gonadotropin
ICF	informed consent form
ICH	International Conference on Harmonisation
ID number	identification number
IEC	Independent Ethics Committee
IRB	Institutional Review Board
ITT	intent-to-treat
IUD	intrauterine devices
MMSE	Mini Mental State Examination
MG	milligram(s)
N	number of observations
NPI	Neuropsychiatric inventory
pH	grade of acidity
PK	pharmacokinetics
PP	per-protocol
QA	quality assurance
QC	quality control
RANUNI	random numbers which have uniform distribution
RCT	randomized clinical trial
SAE	serious adverse event
SAS	statistical analysis system
SCIA	Structured Clinical Interview for Apathy
SOP	standard operating procedure
SS	safety (data) set
TMF	trial master file
TCS	transcranial sonography
UHDRS	Unified Huntington's Disease Rating Scale
UK	United Kingdom
UNL	upper normal limit
WHO	World Health Organisation

PROTOCOL SUMMARY

**Sponsor:**	Charité, Universitätsmedizin - Berlin
**Protocol ID No.:**	HDSY001
**EudraCT No.:**	2009-013698-16
**Title:**	A randomized, double-blind, placebo-controlled prospective crossover trial investigating the efficacy and safety of the treatment with Bupropion in patients with apathy in Huntington's disease
**Clinical Phase:**	II
**Objectives:**	The influence of bupropion compared to placebo on the change of apathy as quantified by the apathy evaluation scale (AES-I, where I [informant] is a friend or family member familiar with the daily activities of the subject) in patients with HD after ten (10) weeks of treatment.
**Study Design:**	A randomized, double-blind, placebo-controlled, crossover, proof of concept phase II study (investigator initiated trial (IIT)
**Number of Subjects:**	40 male and female patients with HD
**Number of Centres:**	4 investigative sites in Germany
**Study Population:**	Patients with HD (moleculargenetic confirmed by CAG repeat prolongation) and apathetic symptoms (SKIA-D).<br><br>**Inclusion Criteria:**<br>1. Verified HD mutation carriers aged 25 to 65 years (inclusive) at first dosing<br>2. Apathetic as diagnosed by SCIA-D criteria<br>3. Stable concomitant medication (no change of medication during last three months prior to inclusion)<br>4. Written informed consent by prospective study participant before conduct of any trial-related procedure. Participant must be able to make an informed decision of whether or not to participate in the study<br>5. Patient has a caregiver (family member or friend), who is living in a close relationship with the patient and is willing to give written informed consent (caregiver) before performance of any trial-related procedure<br><br>**Exclusion criteria:**<br>1. Pregnant or nursing women<br>2. Active suicidality based on the answer "yes" in questions 4 and 5 of the Columbia-Suicide Severity Rating Scale (baseline version)<br>3. Woman of childbearing potential, not using highly effective methods of contraception defined as methods with a Pearl Index < 1 such as oral, topical or injected contraception, IUD, contraceptive vaginal ring, or double barrier method such as diaphragm and condom with

	spermicide) or not surgically sterile (via hysterectomy, ovarectomy or bilateral tubal ligation) or not at least one year post-menopausal<br>4. Male not using an acceptable barrier method for contraception and donating sperm from screening up to three months following treatment<br>5. Presence or history of any medically not controllable disease (e.g. uncontrolled arterial hypertension or diabetes mellitus)<br>6. Presence or history of seizures or diagnosed epilepsy or history of severe head trauma (contusio cerebralis) or CNS tumor<br>7. Clinical significant renal (calculated creatine clearance < 60 ml/min) or hepatic dysfunction<br>8. Clinical significant depression defined by the NPI depression score (score ≥4 points) at screening<br>9. Schizophreniform psychosis within the last 6 months prior to first dose<br>10. History of anorexia or bulimia<br>11. Severe cognitive disorders defined as a score < 18 in the Mini-Mental State Examination (MMSE) at screening<br>12. Marked chorea (UHDRS 4) of face, BOL, trunk or extremities<br>13. Treatment with neuroleptics other than tiaprid, MAO-B inhibitors, amantadine, levodopa, D- or D,L-amphetamine or psychostimulants like methylphenidate, modafinil or atomoxetine within 1 month prior to first dose<br>14. Known hypersensitivity reaction associated with bupropione, gelatine, lactose or magnesium stearate<br>15. Clinically relevant abnormal findings in the ECG, the vitals, in the physical examination or laboratory values at screening that could interfere with the objectives of the study or the safety of the subject as judged by the investigator<br>16. Acute disease state (e.g. nausea, vomiting, fever, diarrhoea, infection) within 7 days of first dose<br>17. Definite or suspected personal history or family history of adverse reactions or hypersensitivity to the trial compounds (or to compounds with a similar structure)<br>18. Presence of illicit drug and/or alcohol abuse<br>19. Participation in another investigative drug trial within 2 months or donation of blood within 12 weeks prior to the first dose or during the trial<br>20. Subjects who are unlikely to be compliant and attend scheduled clinic visits as required<br>21. Placement in an institution due to governmental or judicial authorities
**Investigational medicinal products:**	Bupropion 150 mg/300 mg or placebo
**Duration of treatment:**	**first treatment group:** 150 mg bupropion or placebo once daily for 2 weeks, followed by 300 mg bupropion or placebo once daily for subsequent 8 weeks (until week 10; visit 4)<br>**first tapering and washout:** 150 mg bupropion or placebo once daily for 7 days followed by a washout phase of 1 week on placebo

	**second treatment group (crossover):** placebo or 150 mg bupropion once daily for 2 weeks, followed by placebo or 300 mg bupropion once daily for subsequent 8 weeks (until week 22; visit 6)<br>**second tapering** placebo or 150 mg bupropion once daily for 7 days
**Evaluations:**	**Primary efficacy variable:**<br>The primary efficacy parameter is the AES-I score after ten weeks of treatment adjusted for baseline.<br>The following secondary variables will be analysed:<br>• change of apathy as quantified by the AES-C (clinician) or the AES-S (self),<br>• change of motor symptoms (UHDRS) and quantitative grip force motor assessment,<br>• change of cognitive symptoms (UHDRS and MMSE),<br>• change of psychiatric symptoms (UHDRS, HADS),<br>• change of activities of daily living (UHDRS),<br>• change of the NPI caregivers' distress score,(NPI-D)<br>• change of ventral striatal and ventromedial prefrontal activation in response to a reward paradigm as quantified by fMRI.
**Statistical methods:**	**Efficacy:**<br>The confirmatory inferential statistical evaluation of the primary target parameter will be based on a linear mixed effects model with the AES-I score as dependent variable. This model will use treatment group and period as fixed factor and baseline AES-I score as covariate. Random effects will be introduced for the intercepts and thus taking the correlation within patients into account.<br>All secondary efficacy variables will be evaluated by using descriptive statistics primarily. If applicable, a similar analysis will be performed for secondary variables in an explanatory manner.<br><br>**Safety:**<br>The results of AE recording, clinical laboratory and physical examination collected in the study will be appropriately summarized and analyzed for each treatment group by descriptive statistics using tabulation and graphs.
**Planned trial dates**	01/10/2011 – 01/04/2013

SCHEDULE OF PROCEDURES

	Visit 1	Visit 2	Visit 3	Phone Interview 1	Visit 4	Visit 4a	Visit 5	Phone Interview 2	Visit 6	Visit 7
	Screen -3 weeks +/-1 week	Week 0	Week 3 +/-2 days	Week 4 +/-2 days	Week 11 +/-1 week	Week 13 +/-1 week	Week 15 +/-2 days	Week 16 +/-2 days	Week 23 +/-1 week	FUP[5] +/-1 week
	Day -28 to -14	Day 0	Day 15	Day 22	Day 71	Day 85	Day 99	Day 106	Day 155	Day 169 to 183
Informed consent	X									
In- and exclusion criteria	X	X								
Randomisation		X								
Medical history	X									
Physical examination	X				X				X	X
Concomitant medication	X	X	X		X	X	X		X	X
Vital signs, ECG	X				X	X			X	X[7]
Laboratory	X[1]		X[2]		X[1]	[1]	X[2]		X[1]	X[2]
Pregnancy test[6]	X	X	X		X	X	X		X	
CAG-repeats	X									
Study medication[3]		X	X	X	X	X	X	X	X	X
Adverse events		X	X	X[4]	X	X	X	X[4]	X	X
C-SSRS (baseline)	X									
C-SSRS (since last visit)		X	X	X	X	X	X	X	X	X
AES-I		X	X		X	X	X		X	
AES-S/AES-C		X	X		X	X	X		X	
SCIA-D	X				X	X			X	
NPI (depression/ dysphoria)	X				X	X			X	
NPI-D	X				X	X			X	
HADS-SIS	X				X	X			X	
UHDRS	X				X	X			X	
MMSE	X				X	X			X	
Grip force	X				X	X			X	
fMRI		X			X					

1. Laboratory examinations on visit 1, visit 4 and visit 6 (haematology, clinical chemistry, and urinalysis).
2. Laboratory examinations on visit 3, visit 5 and visit 7 (haematology, clinical chemistry safety monitoring).
3. Dispensing and collection of IMP; Day 1 first dosing; Dosing from day1 to day14: 150 mg/placebo (1 capsule) once daily, from visit 3 (day 15) until visit 4 (day 70): 300 mg/placebo (2 capsules) once daily; from visit 4 (day 71) until visit 4a (day 85) 5 : 150 mg/placebo (1 capsule) for 7 days followed by placebo (1 capsule) once daily for 7 days; from visit 4a until visit 5 (day 98): placebo/150 mg (one capsule) once daily for 14 days (crossover after week 12); from visit 5 (day 99) until visit 6 (day 154): placebo/300 mg (2 capsules) once daily; from visit 6 (day 155) placebo/150 mg (1 capsule) once daily for 7 days.
4. After week 3 and week 14 (one week after dose increase from 150 mg to 300 mg), a phone interview will be conducted: Subjects will be enquired for possible side effects of medication.
5. Visit 7 must be 3 weeks (+/- one week) after visit 6.
6. A serum pregnancy test will be performed at the screening visit and a urine pregnancy test during the subsequent visit(s)
7. ECG and vital signs examination will be performed during Visit 7 if judged necessary for medical reasons by the treating physician after visit 6.

SCHEDULE OF TREATMENT

All subjects will receive Bupropion 150 mg/300 mg or placebo for 23 weeks.

**Visit 2-3:**<br>(week 1 and 2)<br>150 mg bupropion or placebo once daily for 2 weeks	**Visit 3-4:**<br>(week 3-10)<br>300 mg bupropion or placebo once daily for 8 weeks	**Visit 4-5:**<br>(week 11-14)<br>***tapering:*** 150 mg bupropion or placebo for 1 week<br>***washout:*** placebo for 1 week<br>***crossover:*** placebo or 150 mg bupropion once daily for 2 weeks	**Visit 5-6:**<br>(week 15-22)<br>300 mg bupropion or placebo once daily for 8 weeks	**Visit 6-7:**<br>(week 23)<br>**tapering:** 150 mg bupropion or placebo once daily for 1 week

**visit**	1	2	3	ph1	4	4a	5	ph2	6	7
**week**	screen	0	2	3	10	12	14	15	22	FUP

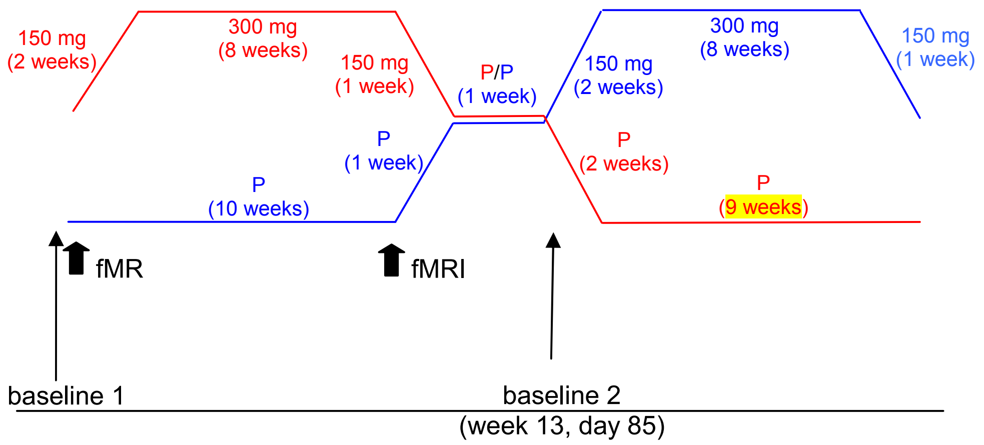

1 INTRODUCTION AND RATIONALE

Morbus Huntington or Huntington's Disease (HD) is an inherited progressive neurodegenerative disorder clinically characterised by involuntary movements, cognitive impairment, as well as psychiatric and behavioural symptoms. At the molecular level HD is caused by an expanded trinucleotide repeat on the chromosome 4 within the coding region of the Huntington gene.

Next to common psychiatric disorders like depression, anxiety disorders and psychosis, behavioural problems like apathy and irritability are frequently described in HD. These psychiatric disorders and behavioural problems have an enormous impact on functional capacity and emotional relations with partners and families.

In fact, apathy was repeatedly ranked as one of the most pressing problems in HD treatment by EHDN investigators.[1] Adequate treatment would alleviate the disease burden, but research about therapeutic options is scarce.

The prevalence of apathy, defined as a primary absence of motivation, lack of initiative, lack of drive and emotional blunting, varies between 34% to 67% in HD.[2,3] Apathy is more often present in advanced stages of HD than in earlier stages, in which irritability and depression are more prevalent.[4,5] In most apathetic patients, motor symptoms are manifest, indicating a relationship with progressive neurodegeneration, in particular in fronto-subcortical circuits.

Functional imaging data suggest that reduction in goal-directed behaviour as a result of loss of motivation may originate in a lack of reward anticipation due to diminished activation of the ventral striatum (VS). This assumption is supported by the observation, that in apathic schizophrenic patients, lower activation of the VS correlated inversely with the AES apathy score.[6]

No data are available on the neuroanatomical correlates of apathy in HD. In Alzheimer's disease, quantification of cortical thinning by MRI based morphometry as well as reduced metabolism by functional imaging studies point to the left ACC and the left lateral OFC as major factors in the pathogenesis of apathy.[7] Post mortem morphometic data in HD found a strong association between major mood symptomatology and cell loss in the ACC [8], however functional imaging data are lacking.

Dopamine, but also norepinephrine modulate motivated and goal directed behaviour. In Alzheimer's and Parkinson's disease, an inverse correlation between dopamine transporter density and apathy has been observed. Thus, a shortage of dopamine in the in the fronto-striatal circuits has been proposed as the key pathophysiological mechanism of apathy.[9]

In clinical practice, patients with apathy-like symptoms are often treated with antidepressants like selective serotonin reuptake inhibitors and/or acetyl cholinesterase inhibitors when dementia is concurrent. Unfortunately, no systematic research on specific treatments for apathy in HD has been performed. Most current pharmacological treatment strategies rely on case-reports and case-series. Recently, some larger studies have been performed with bupropion and methylphenidate, but not in patients with HD.[10,11,12]

Bupropion is an inhibitor of the neuronal uptake of dopamine and norepinephrine and does not inhibit monoamine oxidase or the re-uptake of serotonin. In small series, case descriptions and own observations, Bupropion was effective in treating apathy in neuropsychiatric disorders. An improvement of the dopamine shortage via Bupropion enhancing might improve the apathy of HD patients to a clinically significant extent.

Bupropion (Elontril®) has obtained market authorization for treating depression and for smoking cessation. In this study, the drug will be used for the symptomatic treatment of apathy in patients with HD evaluated on clinical scales.

The clinical diagnosis of apathy in HD is difficult because of overlapping symptoms of depression and the consequences of cognitive impairment.[13,14] The SCIA-D is a structured interview which has been validated in neurodegenerative diseases to assess apathy and to differentiate apathy from depression.[15,16] Determining echogenicity of the raphe nucleus by transcranial ultrasound may be helpful in discriminating depression from apathy in HD. [20]

Rating of apathetic symptoms varies when assessed by either the caregiver or the patient. This interrater variability has been attributed to worsening of cognition as the disease progresses.[17] The AES-I, a caregiver apathy rating scale has been chosen as the quantification tool and the primary endpoint in this trial.

2 STUDY OBJECTIVE

2.1 PRIMARY OBJECTIVE

The influence of Bupropion compared to placebo on the change of apathy as quantified by the apathy evaluation scale (AES-I, where I [informant] is a friend or family member familiar with the daily activities of the subject) in patients with HD after ten weeks of treatment.

2.2 SECONDARY OBJECTIVES

- The safety and tolerability of Bupropion in HD.
- The influence of Bupropion compared to placebo on the:
    - change of apathy as quantified by the AES-C (clinician) or the AES-S (self),
    - change of motor symptoms (UHDRS) and quantitative grip force motor assessment,
    - change of cognitive symptoms (UHDRS and MMSE),
    - change of psychiatric symptoms (UHDRS, HADS),
    - change of activities of daily living (UHDRS),
    - change of the NPI caregivers' distress score (NPI-D),
    - change of ventral striatal and ventromedial prefrontal activation in response to a reward paradigm as quantified by fMRI.

2.3 STUDY DESIGN

This is a randomized, double-blind, placebo-controlled, crossover phase II, investigator initiated trial (IIT).

3 PATIENT SELECTION

3.1 NUMBER OF PATIENTS

A total of 40 eligible subjects will be included. Patients will be recruited from the outpatient clinic for HD patients at the participating study centres.

3.2 INCLUSION CRITERIA

1. Verified HD mutation carriers aged 25 to 65 years (inclusive) at first dosing
2. Apathetic as diagnosed by SCIA-D criteria
3. Stable concomitant medication (no change of medication during last three months prior to inclusion)
4. Written informed consent by prospective study participant before conduct of any trial-related procedure. Participant must be able to make an informed decision of whether or not to participate in the study.
5. Patient has a caregiver (family member or friend), who is living in a close relationship with the patient and is willing to give written informed consent (caregiver) before performance of any trial-related procedure.

3.3 EXCLUSION CRITERIA

1. Pregnant or nursing women
2. Active suicidality based on the answer “yes” in questions 4 and 5 of the Columbia-Suicide Severity Rating Scale (baseline version)
3. Woman with childbearing potential not using highly effective methods of contraception defined as methods with a Pearl Index < 1 (such as oral, topical or injected contraception, IUD, contraceptive vaginal ring, or double barrier method such as diaphragm and condom with spermicide) if they are not surgically sterile (via hysterectomy or bilateral tubal ligation) or not at least one year post-menopausal
4. Male not using an acceptable barrier method for contraception and donating sperm from screening up to three months following treatment
5. Presence or history of any medically not controllable disease (e.g. uncontrolled arterial hypertension or diabetes mellitus)
6. Presence or history of seizures or diagnosed epilepsy, history of severe head trauma (contusio cerebralis) or CNS tumor

7. Clinical significant renal (calculated creatine clearance <60 ml/min) or hepatic dysfunction
8. Clinical significant depression defined by the NPI depression score (score ≥4 points) at screening
9. Schizophreniform psychosis within the last 6 months prior to first dose
10. History of anorexia or bulimia
11. Severe cognitive disorders defined as a score <18 in the Mini-Mental State Examination (MMSE) at screening
12. Marked chorea (UHDRS 4) of face, BOL, trunk or extremities
13. Treatment with neuroleptics other than tiapride, MAO-B inhibitor, amantadine, levodopa, D- or D,L-amphetamine or psychostimulants like methylphenidate, modafinil or atomoxetine within 1 month prior to first dose
14. Known hypersensitivity reaction associated with bupropione gelatine, lactose, magnesium stearate
15. Clinically relevant abnormal findings in the ECG, the vitals, in the physical examination or laboratory values at screening that could interfere with the objectives of the study or the safety of the subject as judged by the investigator
16. Acute disease state (e.g. nausea, vomiting, fever, diarrhoea, infection) within 7 days of first dose
17. Definite or suspected personal history or family history of adverse reactions or hypersensitivity to the trial compounds (or to compounds with a similar structure)
18. Presence of illicit drug and/or alcohol abuse
19. Participation in another investigative drug trial within 2 months or donation of blood within 12 weeks prior to the first dose or during the trial
19. Subjects who are unlikely to be compliant and attend scheduled clinic visits as required
20. Placement in an institution due to governmental or judicial authorities

3.4 SUBJECT COMPLETION AND WITHDRAWAL

3.4.1 Definitions

A 'completed' subject has completed all phases of the study, including any follow-up visit specified.

A ‘screening failure’ is a subject who, after having signed the informed consent, had a screening examination, but who, for any reason, will not be randomized on Day 1 in the trial. Screening failures will not be entered into the database.

A ‘drop-out’ is a subject who stopped prematurely due to non-observance of the protocol or to reasons unrelated to the study, e.g. "moved away" or otherwise lost to follow-up.

A ‘withdrawal’ is a subject who stops prematurely for reasons related to the study, e.g. an adverse event or a treatment failure. A subject who simply wishes to withdraw from the study should also be considered a withdrawal.

In case a serious adverse event related to the study medication occurs, the subject must be withdrawn, unless doing so would harm the patient in the opinion of the investigator.

3.4.2 Procedure for Handling Withdrawals

Subjects withdrawn due to adverse events should be followed-up until the adverse event outcome has subsided or until the condition has stabilized. The study completion/withdrawal section of the eCRF should be completed in any case. Every effort should be made by the investigator to follow-up subjects who have withdrawn from the study.

3.4.3 Removal of Subjects from Therapy or Assessment

All subjects are free to withdraw from participation in this study at any time, for any reason, specified or unspecified, and without penalty or loss of benefits to which the subject is otherwise entitled. Once withdrawn from the study, a subject is not allowed to re-enter the trial.

Subjects may withdraw or be withdrawn for specific reasons before completing the trial. The eCRF has to be completed up to the time of withdrawal and the premature termination form must be completed. Except for subjects who refuse to return to the investigative site, all subjects withdrawn after the first intake of study medication should be given a post-study assessment as appropriate.

The reasons for withdrawal from participation of the study may include the following:

- The subject initiates an early discontinuation:
  - Withdrawal is based on the experience of an adverse event (reason: adverse event).
  - The subject becomes pregnant (reason: other reasons).
  - The subject revoked his/her participation in the study (reason: withdrew consent).
  - Withdrawal is based on other reasons (reason: other reason).
  - The subject fails to return to the site and does not respond to the attempts of the investigative site to contact him (reason: lost to follow up).

- The investigator initiates an early discontinuation:
  - Adverse event, sufficiently severe to contraindicate the continuation of the study (reason: adverse event).
  - Occurrence of seizures, suicidal behaviour or active suicidality based on the answer “yes” in questions 4 and 5 of the Columbia-Suicide Severity Rating Scale (reason: adverse event)
  - Significant intolerance of the study medication or significant clinical laboratory findings e.g. ALAT/ASAT >3.0 x upper reference range or bilirubin >2.0 mg/dl despite prior reduction of study medication from two capsules to one capsule over one week (reason: adverse event)
  - HGB <7,5 g/dl, WBC <2,0/nl, platelets <100/nl, creatinine >250 µmol/l, ASAT/ALAT >5.0 x upper reference range, total bilirubin >3.0 mg/dl (reason: adverse event).
  - Investigator judgement that it is in the best interest of the patient
  - Technical reasons (change of physician, change of address of volunteer/patient)
  - Protocol violation(s), such as non-compliance with study medication or visit schedule or treatment with prohibited concomitant medications (reason: protocol violation).
  - Occurrence of exclusion criteria other than the above named after visit 2

- The sponsor requests the investigator to withdraw the subject from the study
  - for safety reasons (reason: adverse event) or
  - in the event of a significant protocol violation (reason: protocol violation).

3.4.4 Investigator's responsibility following premature discontinuation of patients

For any patient prematurely discontinuing the study, the investigator must:

- In as far as possible, carry out all the examinations and tests planned for the final visit (visit 6) after withdrawal of the subject from the study
- When applicable, ask the patient the reason of her/his withdrawn consent while fully respecting the subject's rights
- Where possible prescribe a visit 2 weeks after withdrawal in order to control and document any change in the patient’s clinical status. If a serious adverse event occurs, the Responsible Safety Contact must be informed according to the procedures described in section 5.4.2.
- When an adverse event is the reason for premature discontinuation, ask the patient to remain under supervision by the clinic until normal conditions are reached or otherwise qualified medical care is guaranteed
- Collect, document and report all safety data until withdrawal

4 STUDY MEDICATION AND ADMINISTRATION

4.1 STUDY MEDICATION

The study medication will be provided by the Pharmacy of the Charité – Universitätsmedizin, Berlin, Germany. Bupropion will be Elontril®, the marketed substance manufactured by GlaxoSmithKline, UK.

4.2 DOSAGE, ADMINISTRATION

Bupropion will be administered in a flexible dosing regimen as proposed by the manufacturer for the treatment of depression. Subjects will be started on a once daily oral dose of 150 mg (one capsule) in the morning. If well tolerated, the dose will be increased to 300 mg (two capsules) once daily after two weeks of treatment. This will be the stable dose throughout the following eight (8) weeks of treatment. One week after start of full dose regimen (week 4) a phone interview will be conducted. In case of side effects after dose increase, which are not tolerable for the patient or could interfere with the safety of the subject as judged by the investigator, the dose can be reduced to 150 mg (one capsule) once daily. A dose reduction that occurs will remain in effect for the duration of treatment. After week 10, 150 mg (one capsule) will be given for seven days to all subjects originally in the verum group before they will be given placebo for one week. Subjects so far receiving placebo will be given another week of placebo, before a once daily oral dose of 150 mg (one capsule) in the morning will be administered for two weeks. If well tolerated, the dose will be increased to 300 mg (two capsules) once daily. This will be the stable dose throughout the following eight (8) weeks of treatment. One week after start of full dose regimen (week 16) a phone interview will be conducted. In case of side effects after dose increase, which are not tolerable for the subject or could interfere with the safety of the subject as judged by the investigator, the dose can be reduced to 150 mg (one capsule) once daily. A dose reduction that occurs will remain in effect for the duration of treatment. After week 22 150 mg (one capsule) will be given for seven days. Subjects who received placebo in the second line will continue the intake for another seven days.

4.3 METHOD OF BLINDING

The trial will be conducted as a randomized, placebo-controlled, double-blind trial (RCT) in four centres in Germany. The Pharmacy of the Charité will encapsulate the bupropion tablets in hard gelatine capsules ensuring the concealment of active or placebo treatment allocation from study site staff and patients. Based on the randomization code provided by the Institute for Medical Statistics, Inforamtics and Documentation, University Hospital Jena (Prof. Dr. Schlattmann) each

centre will be provided with sequentially numbered, tamper-proof containers which are equal in weight and similar in appearance.

4.4 MANUFACTURING AND PACKAGING

Bupropion, Elontril®, will be manufactured by GlaxoSmithKline, UK. The Pharmacy of the Charité, Universitätsmedizin-Berlin will encapsulate the study medication (verum and placebo). The study medication will be packed and labelled by the pharmacy.

Description of Placebo

- excipients: 8 mm white, Lichtenstein
- dosage form: orange-red gelatine capsules size DB AA (Capsugel NV; HGC AADB 22.307/22.307)
- container: HDPE 40 ml (Securipac) Zscheile&Klinger including Tropack mini desiccant bags.
- manufacturer: Pharmacy of the Charité, Universitätsmedizin-Berlin
- batch number: as labeled
- expiry date. as labeled

4.5 LABELLING AND PREPARATION

The IMP will be labelled in accordance with local laws and with the European GMP Guideline Annex 13 (Investigational Medicinal Products). The information on the label will be written in German and will include the following details as a minimum:

• Protocol number	HDSY001
• Patient identification number	Pat.-Nr.:
• EudraCT number	2009-013698-16
• Identifying lot or control number	Ch.-B.:
• Name or code designation of the drug	
• Expiry date	DD-MMM-YYYY
• Dosage form	capsule
• Quantity of contents	7 capsules Bupropion 150 mg or placebo, or
•	21 capsules Bupropion 150 mg or placebo, or
•	64 capsules Bupropion 150 mg or placebo
• Name and address of sponsor	Charité-Universitätsmedizin Berlin<br>Klinik für Psychiatrie und Psychotherapie<br>Charitéplatz 1, 10177 Berlin<br>LKP: Prof. Dr. J. Priller, Tel.: 030 450 6172 39<br>Tel.: +49 (0)30-450 617 239
• Directions for use / dosage	take 1 or 2 capsules once daily before breakfast
• Advice	“store closed container in a dry place at room temperature”<br>“for clinical trial use only”

"Return unused medication
"keep out of reach of children"

4.6 STORAGE

The investigational medicinal product must be stored in a locked dry place at room temperature at the centre before distribution to the individual subject.

4.7 DRUG ACCOUNTABILITY

The principal investigator or his/her designee must maintain precise and accurate records of the disposition of all investigational medicinal products supplied to their centre. At all times during the study it must be possible to control the date of dispensing and the quantity of study medication given to each patient. The investigator must not destroy any label or any box of medication even if empty or only partly used. The investigator or a nominated individual, who has been authorized by the investigator, will have to retain all used and unused medication from the patient. At the end of the clinical trial the monitor will review the drug accountability records before arranging for all used and unused medication to be returned to the Pharmacy of the Charité, which will manage its legal destruction.

4.8 ASSESSMENT OF TREATMENT COMPLIANCE

The investigational medicinal products will be dispensed to patients at visit 2, visit 3, visit 4, visit 5 and visit 6. Patients must return containers with all remaining capsules for compliance check to their next scheduled visit. Returned capsules must be counted and documented at visit 3, visit 4, visit 5, visit 6 and visit 7 for compliance check.

4.9 SAFETY EVALUATIONS

4.9.1 Clinical Laboratory Tests

The investigators will have to evaluate all laboratory values and will have to sign and date the results on the respective print-out. Those values, which are outside the normal range, must be assessed as not clinically significant (NSC) or as clinically significant (CS). Parameters that have been assessed as clinically significant (CS) and /or the corresponding clinical diagnosis shall be documented as adverse events.

4.9.2 For Female Patients

Females of childbearing potential will undergo a serum pregnancy test at the pre-study screening visit and a urine pregnancy test at subsequent study visits. A female is considered to be of

childbearing potential if she possesses a uterus and at least one ovary, is not surgical sterilized (via hysterectomy, ovarectomy or tubal ligation) or is not at least one year postmenopausal.

4.9.3 Adverse Events (AE) of Bupropion

During each visit, patients will be queried indirectly regarding adverse events. Patients will also be questionned to determine if they experienced any change in their mood or experienced any new psychiatric symptoms. Bupropion is licensed for the treatment of major depression and smoking cessation. The adverse event profile is known (for detailed description: see product information).

In clinical trials, the following AEs were seen more often with Bupropion treatment than in placebo treated patients: Abdominal pain, agitation, anxiety, dizziness, dry mouth, insomnia, tremor, myalgia, nausea, vomiting, obstipation, palpitations, pharyngitis, sweating, tinnitus, and changes of urinary frequency, hypersensitiv reactions like urticaria, loss of appetite, headache, taste disorder, and/or impaired vision.

As HD patients have an increased risk of suicide, there has been a long-standing concern that antidepressants (including Bupropion) have a role in inducing or worsening depression and the emergence of suicidality in certain patients during the early phase of the treatment.

All patients should be carefully monitored and observed for clinical worsening, suicidality and unusual changes in behaviour during the initial weeks of the study.

Patients should be monitored carefully for the following symptoms: anxiety, agitation, panic attacks, insomnia, irritability, hostility, aggressiveness, impulsivity, akathisia, hypomania and mania.

Families and caregivers of patients being treated should be informed at screening and baseline about the need to monitor patients for the emergence of agitation, irritability, or unusual changes in behaviour and that they should be asked to report any of these symptoms immediately to the investigator.

In addition, patients will be guided through a semi-structured scale (Columbia-Suicide Severity Rating Scale; C-SSRS) that assesses the full spectrum of suicidal ideation and behavior and monitors changes in symptoms across time.

The frequency of study visits (8 visits in 24 - 27 weeks), especially during the first weeks of dosing, information of study participants and caregivers/informants on possible side effects of the study drug (especially irritability, suicidality and epileptic seizures) allows for early detection of emerging side effects relevant for the safety of the study participant.

4.9.4 Concomitant Medication / Treatment

Required medication for the treatment of concomitant diseases and known not to interfere with the study drug or to mask the effect of the study drug is unrestricted and may be continued during the study as clinically indicated. This includes medication administered for symptomatic treatment in Huntington's disease not excluded unter 3.3. All concomitant medication including over-the-counter medical products products (dietary supplements, herbal medications, etc.) taken during the study must be recorded in the eCRF with indication, route, daily dose and dates of administration and start and termination dates of its administration. The patient should be asked to inform the investigator prior to starting any new medication during the course of the study.

The following concomitant medication/treatment is prohibited during the whole study period: MAO-B inhibitor, amantadin, levodopa and amphetamine or its derivates.

4.9.5 Treatment after Completion of the Study

The therapy of individual patients may be continued after completion of the trial (visit 7) according to the decision of the treating physician.

5 ADVERSE EVENTS

5.1 ELICITING AND DOCUMENTING ADVERSE EVENTS

It is the responsibility of the investigator to document all adverse events which occur during the investigation. An adverse event includes any noxious, pathologic or unintended change in anatomical, physiological or metabolic functions as indicated by physical signs, symptoms and/or laboratory changes occurring in any phase of the clinical study whether associated with study drug or placebo and whether or not considered to be drug related. This includes exacerbations of pre-existing conditions or events, intercurrent illness and suspected drug interactions.

All adverse events occurring after written informed consent must be reported. Subsequent adverse events, irrespective of whether no drug, active drug or placebo has been administered, must be reported regardless of whether or not they are considered to be drug related.

At each assessment, all adverse events either observed by the investigator or one of his clinical collaborators, or reported by the patient spontaneously or in response to a direct question must be evaluated by the investigator and noted in the adverse event sections of the patient’s eCRF.

Any clinically significant changes in laboratory parameters and /or the corresponding clinical diagnosis will be recorded as an adverse event in the relevant section of the eCRF.

The non-leading question "Have you felt different in any way since your last visit?" will be asked at baseline and the question "Do you feel different in any way since starting the new treatment/the last assessment?" will be asked at regular intervals throughout the study. The responses will be recorded in the patient’s eCRF.

The nature of each event, date onset, severity, relationship to treatment, action taken and outcome should be established. Details of changes in the dosage schedule or any corrective treatment should be recorded on the appropriate pages of the eCRF.

5.2 ASSESSMENT OF SEVERITY

Maximum intensity should be assigned to one of the following categories:

Mild: For example, an adverse event which is easily tolerated by the patient, causing minimal discomfort and not interfering with everyday activities.

Moderate: For example, an adverse event which is sufficiently discomforting to interfere with normal everyday activities.

Severe: For example, an adverse event which is incapacitating and prevents normal everyday activities.

The severity does not reflect the clinical seriousness of the event and is evaluated independently from relation to study treatment.

5.3 ASSESSMENT OF CAUSALITY

Every effort should be made by the investigator to explain each adverse event and assess its relationship, if any, to the treatment with the study drug. Causality should be assessed using the following categories: unrelated, unlikely related, possibly related, probably related, definitely related.

UNRELATED: AEs which are judged to be clearly and incontrovertibly due to a cause other than the study drug (concurrent illness etc.).

UNLIKELY RELATED: the temporal sequence is atypical and AE does not follow a known pattern of response to the study drug; another causative factor is present but causal role of the study drug cannot be excluded.

POSSIBLY RELATED: either an event that is temporally associated with the use of the investigational product, a known pharmacological effect/associated reaction but which is also recognised with another concomitant therapy/illness or other external cause.

PROBABLY RELATED: an appropriate temporal association, known pharmacological effect, recognised to be associated investigational product reaction and for which no other possible cause is evident.

DEFINITELY RELATED: the reaction has occurred with this investigational product previously (i.e. positive rechallenge) and there is an appropriate temporal relationship between therapy and reaction; and reactions follows a known or expected response pattern to the suspected drug.

The degree of certainty with which an adverse event is attributed to drug treatment (or alternative causes, e.g. natural history of the underlying diseases, concomitant therapy etc.) will be determined by how well the event can be understood in terms of one or more of the following:

a. known pharmacology of the drug
b. reaction of similar nature previously observed with this drug or this class of drug
c. event having often been reported in the literature for similar drugs as drug related
d. event being related by time to drug ingestion terminating with drug withdrawal (de-challenge) or reproduced on re-challenge.

5.4 SERIOUS ADVERSE EVENTS

5.4.1 Definition of Serious Adverse Events

A serious adverse event is any untoward medical occurrence that at any dose results in death including self-inflicted death or the attempt to do so, is life threatening, requires inpatient hospitalization or prolongation of existing hospitalization, results in persistent or significant disability/incapacity, or a congenital anomaly / birth defect. Any planned or elective surgery or intervention will not be reported as a serious adverse event.

In addition, medical and scientific judgment should be exercised in deciding whether other conditions should also be considered serious, such as important medical events that may not be immediately life threatening or result in death or hospitalization but may jeopardize the patient’s safety or may require intervention to prevent one of the other outcomes listed in the definition above. These should also be considered serious. Examples of such events are intensive treatment in an emergency room or at home for allergic bronchospasm; blood dyscrasias or convulsions that do not result in hospitalization; or development of drug dependency or drug abuse.

Life threatening definition:
An adverse event is life threatening if the patient was at immediate risk of death from the event as it occurred; i.e. it does not include a reaction that if it had occurred in a more serious form it might have caused death. For example, drug induced hepatitis that resolved without evidence of hepatic failure would not be considered life threatening even though drug induced hepatitis can be fatal.

Disability / incapacitating definition:
An adverse event is incapacitating or disabling if the event results in a substantial and/or permanent disruption of the patient's ability to carry out normal life functions.

5.4.2 Reporting of Serious Adverse Events

Any serious adverse event which occurs after signing the informed consent form until the last study visit (VISIT 7- ≤ 28 d after receiving the last treatment with study medication), whether or not related to the study drug, must be reported by the investigator within 24 hours by telephone or fax and, in addition, by e-mail to the following person:

Dr. Wilhelm Fischer,
Klinik für Neurologie, Universität Ulm,
Oberer Eselsberg 45, 89081 Ulm, Germany
phone: +49 / 731-500-63000
fax: +49 / 49 731-500-63002
e-mail: wilhelm.fischer@uniklinik-ulm.de

The investigator should not wait to receive further information before notifying the above person of a serious adverse event. The telephone/fax report should be followed by a full written report on the CIOMS-form, detailing relevant aspects of the adverse event including copies of relevant hospital case records, autopsy reports and other documents where applicable.

5.4.3 Follow-up of Adverse Events

The investigator should follow-up on patients with adverse events until the event has subsided (disappeared) or until the condition has stabilized. Reports relative to the patient’s subsequent course must be submitted to the clinical trial monitor.

5.5 OVERDOSAGE

Any instance of overdose (suspected or confirmed) must be communicated to the sponsor. Details of any signs or symptoms and their management should be recorded including details of any antidote(s) or treatment administered.

5.6 PREGNANCY

Female patients who were tested positive in a pregnancy test during the baseline assessment are not eligible for study participation. Female patients who become pregnant after having received the study medication have to notify the investigator immediately after they have received information about their pregnancy. In case of a positive pregnancy testing, study medication is not allowed and this patient has to be withdrawn. Whenever possible a pregnancy should be followed to term, any premature terminations should be reported, and the status of the mother and child should be reported to the sponsor after delivery. The same procedure shall be followed in cases where the female partner of a male patient becomes pregnant.

5.7 BREAKING THE STUDY BLIND

Only in the case of a serious adverse event, which cannot be adequately treated in the view of the investigator without knowing the identity of the study medication the emergency code can be broken for a particular patient. Every effort must be made to contact the coordinating investigator or the project coordinator as representatives of the sponsor prior to breaking the code. If this is not possible and if the situation is an emergency, the investigator may break the code and contact the coordinating investigator or the project coordinator as representatives of the sponsor as soon as possible thereafter. The breaking of the code should be documented by the investigator in the eCRF, including the reason for doing so, by whom the code was broken and the date on which it was broken.

6 STUDY PROCEDURES

6.1 ASSESSMENTS

The following sections describe in detail all study procedures. A flow chart of all study procedures is presented at the beginning of the protocol. An electronic case report form (eCRF) is provided for each patient. Assessments will be done at the same order during each visit.

6.1.1 Unified Huntington's Disease Rating Scale (UHDRS)

The UHDRS is a score to measure the severity of symptoms associated with HD, subdivided into four subscores including motor, behavioural, cognitive and functional assessment. Motor, behavioural and functional assessments (total functional capacity and functional scale) will be done by the investigator. Cognitive testing (Stroop test, verbal fluency test, symbol digit modalities) will be done by the investigator together with the patient. The UHDRS will be performed at visit 1, visit 4 and visit 6.

6.1.2 Quantitative grip force and speeded tapping motor assessment

The quantitative grip force motor assessment quantifies the coordination of isometric grip forces in the precision grip between the thumb and index finger during grip initiation, object transport and static holding phase. While sitting at a table, the patient grasps the grip device and holds it stable next to a marker at a predefined height. Grip force and object position are measured using a small grip device, equipped with a pre-calibrated force transducer measuring grip (normal) and lift (vertical) forces (a Polhemus 3D position sensor measures x-, y-, z-position and roll-, pitch, yaw-orientation). For completion, speeded tapping (5x10s for right and left index finger will be added). Quantitative grip force and speeded tapping motor assessment will be done at visit 1, visit 4 and visit 6.

6.1.3 SCIA-D: (Structured Clinical Interview for Apathy - Dementia)

The SCIA-D is a structured interview validated to assess apathy in patients with Alzheimers's disease and to differentiate apathy from depression in those subjects by assessing 7 domains relative to the individual's previous level of functioning. Each criterion will be assessed with two key questions, and additional follow up questions are used to rate the severity of symptoms. The SCIA-D will be performed at visit 1, visit 4 and visit 6.

6.1.4 Apathy Evaluation Scale (AES)

The apathy evaluation scale (AES)[24] was constructed to assess apathy in clinical populations by the subject (AES-S), by the clinician (AES-C) and by an informant (AES-I) who is able to observe the subject on a daily basis. The AES scale consists of 18 items, each of which is assessed on a 4-point Likert scale such that the score ranges from 18 to 72. The AES will not be used as an inclusion criterion, as there is no validated cut-off for the presence of clinically relevant apathy in HD. It will be answered at visit 2, visit 3, visit 4, visit 5 and visit 6.

6.1.5 Neuropsychiatric Inventory (NPI)

The neuropsychiatric inventory (NPI)[25] is a well established instrument for the assessment of neuropsychiatric symptoms in neurodegenerative diseases. The NPI is composed of 12 subdomains and is completed by the informant. For each subdomain, the maximum score is 12 (frequency x severity). In the present study, only the subdomains “depression/dysphoria” and “irritability/lability” will be used will be done at visit 1, visit 4 and visit 6. The caregivers’ distress will be rated on a scale from 0 to 5 for each NPI domain. Measurement of the caregivers’ distress is crucial in this trial, because the distress might affect his/her judgement of apathy and influence the AES-I, which is the primary objective of the trial.

6.1.6 Hospital Anxiety and Depression Scale combined with the Snaith Irritability Scale (HADS-SIS)

The HADS is a self reporting questionnaire consisting of 14 items rating symptoms of depression (7 items) and anxiety (7 items) that reflect mood rather than cognitive and somatic symptoms[26]. The SIS rates irritability (8 items), where four items are focused on inwardly and four items on outwardly focused irritability.[27] The HADS-SIS combines the HADS with the Snaith Irritability Scale. Each item is rated on a four-point scale. The HADS-SIS will be answered at visit 1, visit 4 and visit 6.

6.1.7 Columbia-Suicide Severity Rating Scale (C-SSRS)

The C-SSRS is a semi-structured scale that assesses the full spectrum of suicidal ideation and behavior and monitors changes in symptoms across time. It is the only suicide measurement that includes embedded definitions for distinct terms (suicidal ideation, behavior and non-suicidal self-injurious behavior) and provides corresponding probes and anchor points for classifying the severity of suicidal events. The C-SSRS is available in a lifetime history format to assess for suicidal ideation or behavior across the lifespan. A “Since Last Visit” version of the scale assesses for changes since the last administration at every visit including phone interview 1 and 2.

6.1.8 Mini Mental Status Examination

The mini-mental state examination (MMSE) is a brief 30-point questionnaire that is widely used to screen for cognitive impairment, will be performed at visit 1, visit 4 and visit 6. .

6.1.9 Functional MRI

Functional Magnetic resoncance imaging (fMRI) measures the hemodynamic response related to metabolic demand following neural activity in the brain. In this trial, fMRI will be used to measure neural activity in areas thought to be structural and functional correlates of apathy (anterior cingulated, ventral striatum and medial frontal/orbitofrontal cortex) during a reward paradigm with a modified gambling task[28] to measure treatment induced changes in neural activity and functional coupling. Our task consists in a slot machine game, realized in Presentation Control Language (http://www.neurobs.com/), where the rewarding feedback will be the sound of falling coins after a win).

To overcome the problem of involuntary movements, we will use a navigator echo guided, prospective motion corrected multi-echo MPRAGE (vNavs) for structural imaging [29] and partial volume recording (limbic structures and Basalganglia only) in functional imaging to reduce the acquisition time of a single image and thus within scan (not correctable) motion artefacts.

Image processing and analysis will be performed with SPM8 and Freesurfer software packages. Structural algorithms: Voxel Based Morphometry (VBM) of cerebral gray matter (VBM8, http://dbm.neuro.uni-jena.de/) and surface based measures (e.g. cortical thickness). Inference statistics: Analyses of Variance/Covariance (structural & functional data) as well as Psycho-Physiological Interaction (PPI) analyses (functional data), as implemented in SPM8.

Additionally, a resting state measure of 5 minutes duration will take place after the acqusition of the first MPRAGE. The estimated time for one MR session is 35 minutes (retention time into magnet). Functional MRI will be done at visit 2 and visit 4.

The MRI assessments are optional.

6.1.10 12-lead ECG

The ECG will be recorded by means of a 12-lead system for a period of 10 sec while the subjects will rest in the supine position. The chart speed will be set at 25 mm per sec. ECGs will be recorded at screening, visit 4 and visit 6 and 7 if judged necessary for medical reasons after visit 6.

6.1.11 Vital Signs

Systolic and diastolic arterial blood pressure and pulse rate will be measured manually. Measurements will be recorded in the supine position after the subjects have been resting for 3 minutes. Measurement should always be performed at screening, visit 4, visit 6 and FUP (visit 7) on the same arm unless there is a specific contraindication to do this.

6.1.12 Laboratory Methods

Standard methods for clinical laboratory parameters will be used. Documentation of the methods and reference ranges will be part of the clinical file and the study report. Furthermore, the quality certificates being valid during the time of the study will be included into the documentation.

Full laboratory testing (haematology, clinical chemistry, urine alaysis) will be performed during visit 1, visit 4 and visit 6.

Reduced laboratory testing (haematology, clinical chemistry safety monitoring) will be performed during visit 3, visit 5 and visit 7.

A serum pregnancy test will be performed at the pre-study screening visit and a urine pregnancy test during the subsequent study visit(s) for every woman with childbearing potential, who is not surgically sterile (via hysterectomy, ovarectomy or bilateral tubal ligation) or at least one year post-menopausal.

Haematology: Blood (approx. 2 ml) will be placed in an EDTA tube and the following tests will be carried out: HGB, HCT, MCV, MCH, MCHC, RBC, WBC, WBC-DIFF and platelets.

Clinical Chemistry: Blood (approx. 4 ml) will be taken and placed in a serum separation tube and the following parameters will be determined: AP, ALT, AST, GGT, LDH, total bilirubin, creatinine, glucose, sodium, potassium, and additionally albumin, urea-N, cholesterol, triglycerides, total protein.

Clinical chemistry safety monitoring: Blood (approx. 4 ml) will be taken and placed in a serum separation tube and the following parameters will be determined: AP, ALT, AST, GGT, LDH, total bilirubin, creatinine, glucose, sodium, and potassium.

Urinalysis: Urine (approx. 10 ml) will be collected for determination of glucose, pH, ketones, protein, specific gravity.

Pregnancy test: Blood (approx. 4 ml) will be taken at screening visit (visit 1) and placed in a serum separation tube and the following parameters will be determined: ß-HCG. Urine (approx. 10 ml) will be collected to perform a standard urine pregnancy tests at visits 2-6.

CAG-Repeats analysis: For determination of CAG-repeats, 10 ml EDTA plasma will be taken at visit 1 and despatched using the kits provided to BioRep, Milan, Italy for analysis. Results must be available prior to first dosing of the subject.

Blood, serum and urine samples will be sent to and analyzed at the local laboratory of the study site. Urine pregnancy tests will be evaluated by the investigator or an appointed person at the study site. CAG-Repeats analysis will be done by BioRep, Milan, Italy.

6.2 STUDY VISITS

6.2.1 Screening Procedures (Visit 1, week -3 +/- 1 week)

The following procedures have to be performed during screening:

1. Obtaining a signed and dated patient and caregiver informed consent form prior to initiating any study-specific procedure.
2. Assignment of a screening number starting with the number of the study site in an ascending order (e.g. 3-01) to every patient who signs an informed consent form.
3. Review of inclusion and exclusion criteria.
4. Performance of the SCIA-D.
5. Recording of medical history.
6. Recording of drug therapy received or discontinued during the 2 month period prior to the screening visit.
7. Recording of demographics (age, sex and race).
8. Measurement of vital signs (supine blood pressure and pulse rate after three minutes of rest) and recording of an ECG.
9. Collection of blood and urine samples for clinical laboratory tests (haematology, clinical chemistry, determination of CAG-repeats, urinalysis and serum pregnancy testing for every woman with childbearing potential, who is not surgically sterile [via hysterectomy, ovarectomy or bilateral tubal ligation] or at least one year post-menopausal.
10. Performance of a general physical examination (including measurement of weight and height).
11. Performance of UHDRS, Quantitative grip force motor assessment, MMSE and C-SSRS (baseline version) with the patient.
12. Hand out and explanation of the questionnaires to the patient (NPI-depression/dysphoria subscale, HADS-SIS) and the caregiver (NPI-D). It is recommended that following completion

of the questionnaires, the investigator verifies the patient questionnaires to avoid any uncompleted responses.

6.2.2 Visit 2 (Baseline visit; day 0)

The following treatment visit procedures must be performed on day 0 within 2 - 4 weeks following the screening procedures:

1. Review of inclusion and exclusion criteria.
2. Review of concomitant medication. Any changes in medications since the pre-study screening visit should be recorded in the eCRF.
3. Review and recording of adverse events.
4. Performance of a urine pregnancy test if applicable (women with childbearing potential).
5. Performance of the C-SSRS (since last visit version).
6. Hand out and explanation of the questionnaire to the informant/caregiver (AES-I) and the patient (AES-S); after completion, verification of both questionnaires for completeness.
7. Performance of the AES-C.
8. Performance of fMRI examination (optional).
9. Randomization of the patient by assigning the next available randomization number according to the randomization procedure, provided to every centre, hand out of the corresponding study medication and explanation of the dosing schedule to the patient and the informant/caregiver.

During this visit and the following visits, that informant/caregiver has to be the same person.

6.2.3 Visit 3 (2 weeks +/- 2 days after baseline visit [visit 2])

Two weeks +/- 2 days after the baseline visit the patient and the informant/caregiver will be asked to come back to the clinic.

1. Review of the concomitant medication. Any changes in medications since the last visit should be recorded in the eCRF.
2. Review and recording of adverse events.
3. Collection of blood for clinical laboratory tests (haematology, clinical chemistry safety monitoring) and performance of a urine pregnancy test if applicable (women with childbearing potential).
4. Performance of the C-SSRS (since last visit version)

5. Hand out and explanation of the questionnaire to the informant/caregiver (AES-I) and the patient (AES-S); after completion, verification of both questionnaires for completeness.
6. Performance of the AES-C.
7. Collection and recording of all unused medication.
8. Hand out the study medication: In case that no side effects occurred during the preceding 2 weeks, the dose will be increased from 1 capsule to 2 capsules/day (see section 3.9.3).

6.2.4 Phone interview 1 (1 week +/- 2 days after visit 3)

One week +/- 2 days after visit 3, a phone inverview will be conducted with the patient and the informant.

1. Inquire possible side effects of medication. In case of side effects after the dose increase, which are not tolerable for the patient or could interfere with the safety of the subject as judged by the investigator, the dose can be reduced to one capsule/day. A dose reduction will remain in effect for the duration of treatment.
2. Advise the patient and the informant/caregiver on possible side effects and how to report these to the investigator between visits and explain the dosing schedule to the patient and the informant/caregiver.
3. Performance of the C-SSRS (since last visit version).

6.2.5 Visit 4 (8 weeks (+/- 1 week) after visit 3)

Eight weeks (+/- 1 week) after visit 3 the patient and the informant/caregiver will be required to come back to the clinic:

1. Review of the concomitant medication. Any changes in medications since the last visit should be recorded in the eCRF.
2. Review and recording of adverse events.
3. Collection of blood and urine samples for clinical laboratory tests (haematology, clinical chemistry, urinalysis and performance of the urine pregnancy test, if applicable (women with childbearing potential).
4. Measurement of vital signs (supine blood pressure and pulse rate after three minutes of rest) and recording of an ECG.
5. Performance of UHDRS and Quantitative grip force motor assessment with the patient.
6. Performance of the C-SSRS (since last visit version).

7. Hand out and explanation of the questionnaire to the informant/caregiver (AES-I) and the patient (AES-S); after completion, verification of both questionnaires for completeness.
8. Performance of the AES-C.
9. Performance of fMRI examination (if a first fMRI examination has been performed during visit 2 (baseline).
10. Collection and recording of all unused medication.
11. Hand out the study medication and explanation of the dosing schedule to the patient and the informant/caregiver.
12. Performance of SCIA-D and MMSE
13. Hand out and explanation of the questionnaires to the patient (NPI-depression/dysphoria dubscale, HADS-SIS) and the caregiver (NPI-D). It is recommended that following completion of the questionnaires, the investigator verifies the patient questionnaires to avoid any uncompleted responses.

6.2.6 Visit 4a (2 weeks +/- 2 days after visit 4)

Two weeks (+/- 2 days) after visit 4 the patient and the informant/caregiver will be required to come back to the clinic:

1. Review of the concomitant medication. Any changes in medications since the last visit should be recorded in the eCRF.
2. Review and recording of adverse events.
3. Performance of the urine pregnancy test, if applicable (women with childbearing potential).
4. Measurement of vital signs (supine blood pressure and pulse rate after three minutes of rest) and recording of an ECG.
5. Repetition of the dosing schedule of the study medication handed out during visit 4 to the patient and the informant/caregiver.
6. Performance of UHDRS and Quantitative grip force motor assessment with the patient.
7. Performance of the C-SSRS (since last visit version).
8. Hand out and explanation of the questionnaire to the informant/caregiver (AES-I) and the patient (AES-S); after completion, verification of both questionnaires for completeness.
9. Performance of the AES-C.
10. Performance of SCIA-D and MMSE

11. Hand out and explanation of the questionnaires to the patient (NPI-depression/dysphoria dubscale, HADS-SIS) and the caregiver (NPI-D). It is recommended that following completion of the questionnaires, the investigator verifies the patient questionnaires to avoid any uncompleted responses.

6.2.7 Visit 5 (2 weeks +/- 2 days after visit 4a)

Two weeks +/- 2 days after visit 4 the patient and the informant/caregiver will be asked to come back to the clinic.

1. Review of the concomitant medication. Any changes in medications since the last visit should be recorded in the eCRF.
2. Review and recording of adverse events.
3. Collection of blood for clinical laboratory tests (haematology, clinical chemistry safety monitoring) and performance of a urine pregnancy test if applicable (women with childbearing potential).
4. Performance of the C-SSRS (since last visit version).
5. Hand out and explanation of the questionnaire to the informant/caregiver (AES-I) and the patient (AES-S); after completion, verification of both questionnaires for completeness.
6. Performance of the AES-C.
7. Collection and recording of all unused medication.
8. Hand out the study medication: In case that no side effects occurred during the preceding 2 weeks, the dose will be increased from 1 capsule to 2 capsules/day (see section 3.9.3).

6.2.8 Phone interview 2 (1 week +/- 2 days after visit 5)

One week +/- 2 days after visit 5, a phone inverview will be conducted with the patient and the informant.

1. Inquire possible side effects of medication. In case of side effects after the dose increase, which are not tolerable for the patient or could interfere with the safety of the subject as judged by the investigator, the dose can be reduced to one capsule/day. A dose reduction will remain in effect for the duration of treatment.
2. Advise the patient and the informant/caregiver on possible side effects and how to report these to the investigator between visits and explain the dosing schedule to the patient and the informant/caregiver.

3. Performance of the C-SSRS (since last visit version).

6.2.9 Visit 6 (8 weeks (+/- 1 week) after visit 5)

Eight weeks (+/- 1 week) after visit 5 the patient and the informant/caregiver will be asked to come back to the clinic:

1. Review of the concomitant medication. Any changes in medication since the last visit should be recorded in the eCRF.
2. Review and recording of adverse events
3. Measurement of vital signs (supine blood pressure and pulse rate after three minutes of rest) and recording of an ECG.
4. Collection of blood and urine samples for clinical laboratory tests (haematology, clinical chemistry, urinalysis and performance of the urine pregnancy test, if applicable (women with childbearing potential).
5. Performance of a general physical examination (including measurement of weight and height).
6. Performance of UHDRS, Quantitative grip force motor assessment and MMSE with the patient.
7. Performance of the C-SSRS (since last visit version).
8. Performance of SCIA-D with the patient.
9. Hand out and explanation of the questionnaires to the patient (NPI-depression/dysphoria dubscale, HADS-SIS) and the caregiver (NPI-D); after completion, verification of questionnaires for completeness prior to handout of the following questionnaire.
10. Hand out and explanation of the questionnaire to the informant/caregiver (AES-I) and the patient (AES-S); after completion, verification of both questionnaires for completeness.
11. Performance of the AES-C.
12. Collection and recording of all unused medication.
13. Hand out the study medication. Patients will be dosed one capsule 150mg Bupropion/placebo for 7 days. Explain the dosing schedule to the patient and the informant/caregiver.

6.2.10 Visit 7 (Follow-up 3 weeks +/- 1 week after visit 6)

Three weeks (+/- 1 week) after visit 5 the patient and the informant/caregiver will be asked to come back to the clinic:

1. Review of the concomitant medication. Any changes in medication since the last visit should be recorded in the eCRF.
2. Review and recording of adverse events. In case of ongoing AEs, blood will be collected for clinical laboratory tests (haematology, clinical chemistry safety monitoring) if applicable. In women with childbearing potential, a urine pregnancy test will be performed.
3. Measurement of vital signs (supine blood pressure and pulse rate after three minutes of rest) and recording of an ECG, if the ECG recording done at visit 6 showed signs of pathology.
4. Perform a general physical examination (including measurement of weight and height).
5. Collection and recording of all unused medication.

6.3 DATA COLLECTION

Data collected on each patient will be recorded on an electronic case report form (eCRF). The eCRF will be based on the EHDN platform approved by Ethics Committee in the participant centres for the REGISTRY trial of the EHDN (REGISTRY is a multi-centre, multi-national observational study in Huntington's disease with no experimental treatment). The investigator is responsible for ensuring that all sections of each eCRF are completed correctly, and that entries can be verified against source data except for those data, if any, which are to be entered directly in the eCRF. All data derived from measurements during a clinical visit will be entered directly into the eCRF. Laboratory print-outs, external clinical findings or entrances into the patient's medical records etc. (which have to remain at the study site and which will not be collected) are considered as source documents and eCRFs have to be checked against them. If certain data are not available or not applicable, this will be indicated as such in the appropriate space on the eCRF. Any errors and corrections are saved in the audit trial of the EHDN. The study monitor will review the eCRFs and check them for completeness, consistency and plausibility. In addition, any laboratory print-out obtained during the course of the study will have to be inserted manually into the eCRF even if none of the measurements are out of the normal range.

6.4 STUDY MONITORING

The sponsor of this study is responsible according to ICH GCP guidelines for assuring proper study conduct with regard to protocol adherence and validity of the data recorded on the eCRFs. The EHDN has therefore assigned study monitors to this study. Their duties are to assist the investigator in the maintenance of complete, legible, well-organized, and easily retrievable data. In addition, the monitor will ensure that the investigator understands all applicable regulations concerning the clinical evaluation of the investigational drug, as laid down in ICH GCP guidelines. The investigator agrees to allow the monitor access to the study drug dispensing and storage area and to all clinical data of the study patients for the above purposes and agrees to assist the

monitor in these activities. The investigator accepts that the monitor will visit the clinic at regular intervals to review and verify the data collected. The monitor will regard all information which is supplied to him or her as strictly confidential.

6.5 DATA MANAGEMENT

The eCRFs data entry will be checked using computerized and manual means to identify problem fields. Queries will be issued, e.g. on missing data, inconsistencies, illegal values and improperly corrected items (e.g. without initials, date of change or a clear reason for the change). Answers to queries, which have to be digitally signed by the investigator, will be implemented in the database.

Medication names and adverse events will be coded using internationally recognized reference lists (e.g. MedDRA, WHO. DD). Laboratory data will be checked against the normal ranges of the laboratory performing investigation for each centre. Quality control on the data will be performed on an ongoing basis during the study. Clean data sets will be provided to the statistician to perform the statistical analysis.

7 STATISTICAL METHODS AND DETERMINATION OF SAMPLE SIZE

The Department of of Medical Statistics, Informatics and Documentation, University Hospital Jena will be responsible for the statistical analysis of the present trial. Statistical programming and analyses will be performed using the statistical software system SAS® and/or other statistical software as required. A more detailed elaboration of the principal statistical features stated in this protocol will be provided in the statistical analysis plan, which will be finalized prior to unblinding of the database

7.1 SAMPLE SIZE AND POWER CALCULATIONS

This confirmatory study is a multi-centre, randomized, double-blind, placebo-controlled, crossover proof of concept phase II study. The primary objective is to investigate the influence of Bupropion compared to placebo on apathy as quantified by the Apathy Evaluation Scale (AES-I; range 18-72 points) in patients with HD after ten weeks of treatment. Primary efficacy variable is AES-I score after ten weeks of treatment adjusted for the baseline values at week 0 or 13 respectively.

One null-hypothesis will be tested in the confirmatory analysis:

H_0: $\mu_{Bupropion} = \mu_{Placebo}$ vs. H_1: $\mu_{Bupropion} \neq \mu_{Placebo}$,

where $\mu_{Bupropion}$ or $\mu_{Placebo}$ denote the mean change of the AES-I-score after twelve weeks of treatment with Bupropion or placebo.

Earlier research of the Cambridge study site (unpublished data) with the Apathy Evaluation Scale showed a mean score of 31 points (standard deviation SD=15.6). If we define a clinical significant improvement on the AES-I as a 35% reduction of the mean score, this leads to an absolute effect size of 0.35*31 points=10.85 points. Thus a conservative estimate of 10 units is used for sample size estimation. Furthermore a within subjects SD=15.0 is assumed.

When the sample size in each sequence group is 19, (a total sample size of 38) a 2 x 2 crossover design will have 80% power to detect a difference in means of 10,000 (the difference between a Treatment 1 mean, μ_1, of 31 and a Treatment 2 mean, μ_2, of 21) assuming that the crossover ANOVA $\sqrt{MSE}$ is 15,000 (the Standard deviation of differences, σ_d, is 21,213) using a two group t-test (Crossover ANOVA) with a 0,050 two-sided significance level.

In order to account for potential drop-outs 40 patients will be randomized. Sample size calculation was performed with nQuery 7.0

7.2 METHOD OF RANDOMIZATION

Patient allocation to the treatment arms will be based on a central randomization code generated by means of the SAS procedure Proc PLAN provided by the biometrician in charge of the project. The biometrician will have no involvement with the operational aspects of the study or study data until after the database is locked and the blind is broken. No study personnel will have access to this code except for specifically defined emergency situations and the procedure for unblinding relevant patients and who is responsible for that needs to be very clearly defined. The randomization will be based on a permuted block procedure in order to achieve a balanced design. A fixed block size will be used. The randomization will be stratified by centre in order to achieve replication of the 'same' experiment.

7.3 STATISTICAL EVALUATIONS

7.3.1 General Plan

The primary statistical analysis is the testing of the effect of Bupropion in comparison to placebo in patients with HD after ten weeks of treatment within a 2x2 crossover design. The primary efficacy variable is the AES-I score after ten weeks of treatment adjusted for baseline In order to avoid a potential cross-level bias, baseline 1 will be used for each treatment period.

The confirmatory inferential statistical evaluation of the primary target parameter will be based on a linear mixed effects model with the AES-I score as dependent variable. This model will use treatment group (with two levels) and period as fixed factor and the baseline AES-I score as covariate. Random effects will be introduced for the intercepts and thus taking the correlation within patients into account.

Data from all clinical assessments, whether explicitly referred to in the statistics section or not, will be presented in summary tables and in individual patient data listings. Data will be summarised with respect to demographic and baseline characteristics, efficacy and safety observations and measurements.

Standard descriptive summary statistics will be calculated for continuous variables (i.e. arithmetic mean, standard deviation, minimum value, lower quartile, median, upper quartile, maximum value, number of non-missing values). Categorical data will be presented in frequency tables using counts and percentages. Individual patient data listings will be presented parameter-wise and will be sorted by treatment group, centre, patient number and visit. Summary tables will be displayed by treatment group and for the total of the sample. This will be done for each treatment period..

7.3.2 Analysis Sets

The following data sets will be analyzed.

- All patients treated set:

All patients who received at least one dose of study medication will be included in the all patients treated set.

- Full analysis set:

The full analysis set will consist of all patients in the all patients treated set, who had a baseline value and at least one post-baseline assessment for the primary study endpoint.

- Per-protocol analysis set:

The per protocol analysis set will consist of all patients in the full analysis set terminating the study without any major violation of the protocol and its procedures. Patients with major protocol violations will be excluded from the per-protocol analysis.

All analyses with respect to safety and tolerance will be performed for the all patients treated set. All efficacy analyses will be conducted for the full analysis set. Additional efficacy analyses will be done for the per-protocol set in order to obtain efficacy estimates of patients, which more closely reflect the scientific model underlying the protocol. The primary data set for the confirmatory efficacy analysis will be the full analysis set.

Each patient's allocation to the different analysis sets will be identified and mutually agreed on at the “Blind Data Review Meeting” prior to database lock. Deviations from the protocol including violations of inclusion/exclusion criteria will be assessed as ”minor” or ”major” based on the combined decisions of the reviewers according to the specifications outlined in the statistical analysis plan. Major deviations from the protocol will lead to the exclusion of a patient from the per-protocol analysis set. Listings will be prepared to show the eligibility of all patients for the different analysis sets and a summary will be given on the number of patients per analysis set. A more detailed description of the criteria used for data sort out will be included in the statistical analysis plan prior to start of the analyses and before unblinding of the treatment allocation.

7.3.3 Background and demographic characteristics

Assessments done at screening (V1) and baseline (V2) will be displayed in summary tables. These assessments will include demographic characteristics and other relevant parameters (such as medical history and physical examination).

7.3.4 Study medication

Compliance with respect to study medication will be computed based on the available data of the amount of dispensed and returned study medication and the number of days the patient should have taken medication (dispensing intervals).

7.3.5 Concomitant and Pre-study Medications

Concomitant and pre-study medications will be coded according to the WHO terminology using the latest version of the WHO-DRL dictionary, which is available at the time when coding is started. The use of concomitant and pre-study medications will be tabulated by generic drug name and Anatomical Therapeutic Chemical (ATC) code.

7.3.6 Efficacy Analyses

7.3.6.1 Primary efficacy variable

The primary efficacy variable is the AES-I score after ten weeks of treatment adjusted for baseline (baseline 1).

In order to perform a sensitivity analysis, patients who terminated treatment before end of week ten will be imputed using multiple imputation methods in order to substitute missing values. Here, multiple copies of the original dataset will be generated by replacing missing values using e.g. a multivariate normal distribution. Each of these complete sets wil be analysed as complete sets and the corresponding parameter and standard error estimates will be combined with taking the uncertainty of the imputation process into account.

Details will be given in a statistical analysis plan (SAP) together with seed of the pseudo random number generator used for multiple imputation.

Absolute values and changes from baseline will be presented for the AES-I score after ten weeks of treatment using descriptive summary statistics for each treatment period. Moreover percentage changes from baseline will be displayed.

One null-hypothesis as outlined in section 7.1 will be tested in the confirmatory analysis. A linear mixed effect model with treatment and period and baseline values as fixed effects will be used to test this hypothesis. Estimates of means and associated 95% confidence intervals by treatment and treatment period will be provided.

Additionally, the analysis will be adjusted for centre by means of an additional random effect in the model.

The confirmatory analysis of the primary efficacy variable will be done for the full analysis set. Additional efficacy analyses will be conducted for the per-protocol analysis set. The p-values of these analyses will be interpreted in the exploratory sense only.

7.3.6.2 Secondary efficacy variables

The following secondary variables will be analysed;

- change of apathy as quantified by the AES-C (clinician) or the AES-S (self),
- change of motor symptoms (UHDRS) and quantitative grip force motor assessment
- change of cognitive symptoms (UHDRS and MMSE),
- change of psychiatric symptoms (UHDRS, HADS and NPI-D),
- change of activities of daily living (UHDRS),
- change of the NPI caregivers' distress score,
- change of ventral striatal and ventromedial prefrontal activation in response to a reward paradigm as quantified by fMRI.

Analyses for the secondary efficacy variables will be performed for the full analysis set as well as for the per-protocol set. All inferential analyses for the secondary efficacy variables will be interpreted in the exploratory sense.

All secondary efficacy variables will be evaluated by using descriptive statistics primarily (absolute values at each time point and if appropriate changes from baseline).

These analyses will be performed in a similar way as the primary efficacy analysis using a linear mixed effects mode when appropriate.

Image data analysis will be based on standard procedures. Data analysis will be performed using Statistical Parametric Mapping (SPM8; Wellcome Trust Centre for Neuroimaging, London, UK). All images will be corrected for artifacts, transformed into a standard reference space (Internationl Consortion for Brain Mapping – ICBM; http://www.loni.ucla.edu/ICBM/) and spatially smoothed.

Statistical analysis will be performed in a two-stage linear mixed effects model:
(i) The first stage consits in single-subject, general linear model (GLM) analyses of stimulus-related brain activity. The GLM contained separate regressors for the experimental conditions (box car functions at stimulus onset, convolved with the canonical hemodynamic response function), covariates of no interest for the six rigid-body-movement parameters determined from motion correction, and single constant representing the mean over scans. Parameters were estimated by a Restricted Maximum Likelihood (ReML) fit.
(ii) In the second stage, we will submit single subjects' contrasts of parameter estimates for the contrast of interest (win anticipation vs. no anticipation) to second level random effects analyses.

A 2 x 2 Analysis of Variance for repeated measures will be computed for the between subject factor TREATMENT (Verum / Placebo) and the within subject factor REPETITION (T1/T2).

To test our a priori hypothesis within the ventral striatum and ventromedial prefrontal cortex, we will compute literature based probabilistic ROIs for a small volume correction (SVC). Spatial coordinates for computation of these ROIs were taken from fMRI publications using comparable reward designs. Based on this data set, we create the ROIs in a three-step process.

(1) The probability that a voxel at a given position lay within the area xxx was estimated by calculating a 3D normal (Gaussian) distribution G(x, y, z) as follows (Turkeltaub et al. 2002):

$$G(x,y,z) = \frac{1}{2\pi\sqrt{|Det(C)|}} exp\left(-\frac{1}{2}[x-\bar{x} \quad y-\bar{y} \quad z-\bar{z}]C^{-1}\begin{bmatrix} x-\bar{x} \\ y-\bar{y} \\ z-\bar{z} \end{bmatrix}\right)$$

where C is the covariance matrix for all coordinate triples x, y, z from the underlying literature and x, y, z are the mean values of the x, y, and z coordinates, respectively.

(2) Because the resulting distribution also contains voxels located in white matter and extracerebral space, we restrict the 3D distribution only to those voxels, which belong to gray matter with a probability of at least 50 percent. To this end we used the gray matter probability map as provided by SPM8.

(3) The outer limits of the finally used ROI were defined by a threshold of threshold SD of the resulting 3D distribution. Finally a binary mask including all surviving voxels was formed.

7.3.7 Safety Analyses

The all patients treated analysis set will be used to evaluate all issues of safety. Safety will mainly be assessed from the incidence of adverse events and the number of laboratory values lying outside predetermined ranges. Other safety data (e.g., ECG, vital signs, and specific tests) will only be taken into account where necessary.

7.3.7.1 Adverse Events

Adverse events will be coded and summarized by the MedDRA code (system organ class and preferred terms). The incidence of adverse events (percent of patients reporting the adverse event at least once) will be calculated by dividing the number of patients who experienced an adverse event by the total number of patients who were administered the respective treatment. A separate tabulation of incidences will display adverse events stratified by severity (mild, moderate, severe) and by relation to study treatment (unrelated or related according to the respective categories).

The summary tables of adverse events will be broken down by treatment, period, system organ class and preferred term, sorted by total incidence order of each system organ class and each preferred term within each system organ class.

7.3.7.2 Laboratory Data

Laboratory data will be collected at visit 1, visit 4 and at visit 6 (haematology, clinical chemistry, urinalysis) and visit 3, visit 5 and at visit 7, if applicable (haematology, clinical chemistry safety monitoring).

All laboratory values will be converted parameter-wise into most common units (e.g. SI units) for computation of summary statistics, if necessary. Laboratory data will be presented in the listings both in the measured units and in the most common units, if different. Tabulations will use only the most common units. Values outside the investigator’s normal range will be flagged as H (above the normal range) or L (below the normal range) in the listings. Values outside the normal range assessed as clinically significant by the Investigator will be listed separately. For each continuous laboratory parameter, descriptive statistics will be calculated by treatment, period and time of assessment. Shift tables (summarizing changes from baseline to post-study) will be generated. Categorical parameters will be summarized by means of frequency tables by treatment and time of assessment.

7.3.7.3 Vital Signs and Physical Examination Data

Actual measurements of vital signs and findings of the physical examination recorded at visit 1, visit 4, visit 6 and visit 7 (if applicable) respectively, will be listed on an individual basis. Vital signs will be summarized by descriptive statistics by treatment, period and time of assessment. For each visit, summary statistics for the absolute vital signs and the changes from baseline will be presented by treatment group.

Any clinically significant changes in physical examination findings will be discussed in the study report.

7.4 INTERIM ANALYSIS

No interim analysis will be performed.

7.5 FINAL STUDY REPORT

A final study report will be prepared as commitment to Good Clinical Practice. The report will be a record of the total study conduct and will be signed by the Principal Investigators.

8 ETHICS AND INSURANCE

8.1 ETHICAL CONDUCT OF THE STUDY

The study will be conducted in accordance with the EU Clinical Trial Directive 2005/28/EC, the International Conference on Harmonization (ICH) guideline for Good Clinical Practice (GCP) dated July 1996 and the ethical principles laid down in the Declaration of Helsinki (Sommerset West, 1996). Current national regulations and guidelines will also be followed.

8.2 INVESTIGATOR OBLIGATIONS

The Investigator agrees to conduct the clinical study in compliance with the protocol agreed by the Sponsor and, if required by regulatory authorities, which was approved by an Ethics Committee and for which a Clinical Trial Authorization (CTA) has been granted by the Competent Authority (CA). The Investigator and the Sponsor should sign the protocol (and protocol amendments) to confirm this agreement.

The Ethics Committee of the Investigator as well as the CA must review and give approval for the clinical trial as described in this protocol to be conducted in human subjects.

8.3 INDEPENDENT ETHICS COMMITTEE AND COMPETENT AUTHORITY

The study will start only after written approval from an Independent Ethics Committee (IEC) and after obtaining CTA from the CA. The IEC should operate according to ICH GCP guidelines and local law. The written IEC approval and the names and qualifications of members of the IEC must be made available to the sponsor before the study can start. The investigator is responsible for submission(s) to, and communications with, the IEC.

This study protocol (including all substantial amendments) together with the written informed consent form and informed consent updates, subject recruitment procedures (e.g. advertisements), any other written information to be provided to subjects, and any other documents that the ethics committee may need to fulfil its responsibilities will be submitted for approval to the IEC.

The Sponsor should submit written reports of the clinical study status to the IEC annually (Annual Safety Report) and Compentent Authority (CA), or more frequently if requested by the authorities. A final study notification should be forwarded by the sponsor to the Ethics Committee and the CA within one year after the study has been completed.

It is the responsibility of the sponsor to inform all Ethics Committees and responsible national health authorities of all serious adverse events/reactions/SUSARs according to the EU Clinical Trial Directive and according to national provisions.

8.4 SUBJECT INFORMATION AND CONSENT

The subjects will be informed about the nature and importance of the study; they will receive a brief description of the foreseeable risks, discomforts and benefits and a short description of the procedures to be followed. The subjects will be informed that participation in the study is voluntary and that they are free to withdraw from the study at any time without any disadvantages. Prior to the start of the study the subjects will agree to the participation in the study by dating and signing the informed consent form.

Voluntary written informed consent will be obtained from each participant and her/his caregiver (family member or friend, who is living in a close relationship with the patient) at screening prior to any study related procedures. Each subject should be given both verbal and written information (in a language that is understandable to the subject) describing the meaning, aim and conduct of the study. This will take place under conditions where the participant has adequate time to consider the risks and benefits associated with his participation in the study. The subjects will have the possibility to ask all their questions. The consent will be signed and dated by the subject and the person who conducted the informed consent discussion.

It is the responsibility of the investigator to assure that informed consent is obtained from each participant in accordance with Chapter 4.8 of the ICH consolidated guideline for Good Clinical Practice from July 1996, and local regulations. The signed informed consent will be retained with the study records. Each participant will receive a copy of the signed informed consent.

The Investigator should maintain a log of all subjects who sign the informed consent form and indicate if the subject received study drug or, if not, the reason why. The subject's medical records should also document that the informed consent form was signed and dated prior to any study-related procedures being performed.

8.5 INSURANCE

Every subject is insured in accordance with the local laws against damage to health which might occur during the conduct of study and the material damage which occur in connection thereto.

The subject insurance and travel insurance (if appropriate) is taken by the Sponsor. A sample of the insurance policy and insurance conditions is provided to the principal investigator. Contact details of the insurance company are stated in the subject information form.

8.6 RISK-BENEFIT-ANALYSIS

Curative or course-modifying therapies for HD are not available yet. Symptomatic treatment often is of minor effectiveness and hampered by side effects of prescribed drugs. Statistically informative clinical trials on the symptomatic treatment of neuropsychiatric symptoms in HD are not available. In a recent study, apathy was ranked as one of the most pressing problems in HD treatment by EHDN investigators.

Small clinical trials, case studies and own observations firmly suggest the effectiveness of Bupropion in alleviating symptoms of apathy in HD. Evidence based therapeutic alternatives are not available. Following reports on the effectiveness of bupropion in influencing symptoms of apathy in another neurodegenerative disese, Alzheimers Disease, a randomized, BMBF-financed clinical trial is under way.

Thus, patients participating in this study may profit through the reduction of symptoms associated with apathy. They will be seen repeatedly during the course of the study. Medication side effects will likely be detected early, especially when considering present custom of "off-label" prescription of bupropion due to the lack of therapeutic alternatives.

Risks for the participants include complications of venous puncture when drawing blood and possible accidents of the participants and their companions during their journey to the study centre.

For nearly ten years, Bupropion is widely used in the treatment of major depression and smoking cessation. The spectrum of side effects is wellknown. Side effects relevant for the treatment of patients with HD are an emergence or increase in irriatability and suicide ideations. For this, we included instruments for the detection and assessment of irritability and suicide ideations. Especially the C-SSRS will be applied during every study visit and phone contact.

We firmly believe, that the lack of effective treatment for apathy in HD, which so far resulted in therapeutic nihilism, and the availability of a pontentially effective drug requires the evaluation of its effectivens in a potentially statistically informative trial. The possible benefits for the community of HD patients outweighs the possible risks. All Participants in the study will be seen in EHDN-centres by clinicians experienced in the treatment of HD-patients further reducing possible risks for the participants.

8.7 DISCONTINUATION OF THE STUDY

Certain circumstances may lead to early termination of an entire study, in particular for ethical or safety reasons such as:

- The high frequency and/or unexpected severity of adverse events

- Unsatisfactory recruitment of patients in as far as their quantity or quality is concerned or recurrent incomplete/inappropriate collection of data

The decision to prematurely terminate the study will be taken by the sponsor and the coordinating principal investigator.

9 USE OF INFORMATION AND PUBLICATION

It is intended that the results of the study may be published in the scientific literature. Results may also be used in submissions to regulatory authorities. The following conditions are to protect commercial confidential materials (patents, etc), not to restrict publication.

In order to allow for the use of information derived from this clinical study, the investigator understands that he/she has an obligation to provide the sponsor with complete test results and all data developed during this study.

A publication or presentation of the study results may be planned and initiated by the sponsor or his representative. Prior to this, no individual of the scientific personnel involved in this clinical trial, i. e., investigators, co-investigators, EHDN or Sponsor centre will publish or present data or results derived from this study. Together with the principal investigators of each center, the sponsor or his representative will decide on the content of any publication derived from the study. Each participating party of this study should have sufficient time in which to review and comment upon the prepublication manuscript.

In accordance with generally recognized principles of scientific collaboration, co-authorship will be discussed and mutually agreed upon before submission of a manuscript to a publisher. At study start the sponsor will sign an agreement on the publication of the study results with all principal investigators involved in the trial.

10 ARCHIVING OF DATA / RECORDS OF STUDY

The investigator should retain essential documents until at least 2 years after the last approval of a marketing application in an International Conference of Harmonisation (ICH) region and until there are no pending or contemplated marketing applications in an ICH region or at least 2 years have elapsed since the formal discontinuation of clinical development of the investigational product. These documents should be retained for a longer period, however, if required by the applicable regulatory authority.

It is the responsibility of the Sponsor to inform the investigator as to when these documents no longer need to be retained.

Patient files and other source data must be kept for the maximum period of time permitted by the hospital, institution or private practice, but not less than 10 years.

Study documents will include the following:

- Case report forms (eCRFs)
- Data correction forms
- Source documents
- Monitor reports
- Appointment agendas
- Correspondence between sponsor and investigator
- Regulatory documents such as:
  - Signed protocol and any amendments.
  - IEC/IRB approval and other letters from this committee
  - *Curriculum Vitae* of medical personnel (investigators, co-investigators, nurses etc.)
  - Patient Signed Consent forms
  - Investigator’s convention and agreement
  - Study treatment accounting forms

The investigator is responsible for keeping the source documents, i.e. the medical file for each patient participating in the trial, with at least study number and title marked on them.

11 AMENDMENTS TO THE PROTOCOL

Modifications to the protocol should be made as an amendment. These will form an integral part of the protocol and will be signed by the relevant personnel of the sponsor and by the investigator(s).

Substantial amendments should be approved by the Ethics Committee and, where necessary, the competent authority prior to being implemented, unless the amendment is made to eliminate an immediate hazard to the clinical study subjects.

Amendments are regarded as substantial where they are likely to have a significant impact on:

- the safety or physical or mental integrity of the subjects
- the scientific value of the trial
- the quality or safety of any investigational medicinal product used in the trial

Minor changes in the conduct or management of the trial (e.g. change of the monitor etc.) should be notified to the Ethics Committee but will not require Ethics Committee approval unless requested by the Ethics Committee.

If an amendment substantially alters the study design, increases the potential risk to the subjects or affects the treatment of the subject then the information sheet must be revised and submitted to the relevant ethics committee and, where necessary, to the relevant competent authorities, for review and approval. When a subject is currently undergoing study procedures and is affected by the amendment then the subject must be asked to consent again using the new information sheet. The new information sheet must be used to obtain consent from new subjects before enrolment and before the new procedures are performed.

12 REFERENCES

1. Minutes of the EHDN Symptomatic Treatment Working Group Meetings. www.euro-hd.net/html/network/groups/symopt-rx/design/docs/
2. Marin RS: Apathy: a neuropsychiatric syndrome. J Neuropsychiatry Clin Neurosci 1991;3:243-252
3. van Duijn E, Kingma EM, van der Mast RC: Psychopathology in verified Huntington’s disease gene carriers. J Neuropsychiatry Clin Neurosci, 2007;19:441-448
4. Craufurd D, Thompson JC, Snowden JS: Behavioural changes in Huntington’s disease. Neuropsychiatry Neuropsychol Behav Neurol 2001;14:219-226
5. Kingma, EM, van Duijn E, Timman R, van der Mast RC, Roos RAC: Behavioural problems in Huntington's disease using the Problem Behaviours Assessment. Gen Hosp Psychiatry 2008;30:155-161
6. Simon, JJ, Biller, Walther S, Roesch-Ely D, Stippich C, Weisbrod M, Kaiser S: Neural correlates of reward processing in schizophrenia--relationship to apathy and depression. Schizophr Res. 2010 May;118(1-3):154-61
7. Tunnard C, Whitehead D, Hurt C, Wahlund L, Mecocci P, Tsolaki M, Vellas B, Spenger C, Kłoszewska I, Soininen H, Lovestone S, Simmons A: Apathy and cortical atrophy in Alzheimer's disease. Int J Geriatr Psychiatry. 2010 Sep 27. [Epub ahead of print]
8. Thu DC, Oorschot DE, Tippett LJ, Nana AL, Hogg VM, Synek BJ, Luthi-Carter R, Waldvogel HJ, Faull RL: Cell loss in the motor and cingulate cortex correlates with symptomatology in Huntington's disease. Brain. 2010 Apr;133(Pt 4):1094-110.
9. Arciniegas DB, Beresford TP: Diminished motivation and apathy. In: Arciniegas DB, Beresford TP eds. Neuropsychiatry. 1ste ed. Cambridge, Cambridge University Press, 2001, p. 261-283
10. Corcoran C, Wong ML, O’Keane V: Bupropion in the management of apathy. Journal of Psychopharmacology 2004;18:133-135
11. Padala PR, Burke WJ, Bhatia SC, Petty F: Treatment of apathy with methylphenidate. J Neuropsychiatry Clin Neurosci 2007;19:81-83
12. Roth RM, Flashman LA, AcAllister TW: Apathy and its treatment. Curr Treat Options Neurol 2007;9:363-370
13. Levy ML, Cummings JL, Fairbanks LA, Masterman D, Miller BL, Craig AH, Paulsen JS, Litvan I: Apathy is not depression. J Neuropsychiatr Clin Neurosci 1998;10:314-319
14. Marin RS: Differential diagnosis and classification of apathy. Am J Psychiatry 1990;147:22-30
15. Starkstein SE, Petrarca G, Chemerinski E, Kremer J: Syndromic validity of apathy in Alzheimer’s disease. Am J Psychiatry 2001;158:872-877

16. Starkstein SE, Jorge R, Mizrahi R, Robinson RG: A prospective longitudinal study of apathy in Alzheimer's disease. J Neurol Neurosurg Psychiatry 2006;77:8-11
17. Chatterjee A, Anderson KE, Moskowitz CB, Hauser WA, Marder KS: A comparison of self-report and caregiver assessment of depression, apathy, and irritability in Huntington's disease. J Neuropsychiatry Clin Neurosci 2005;17:378-383
18. CPMP Points to Consider on Multiplicity Issues in Clinical Trials 2002
19. Borm GF, Fransen J, Lemmens WA: A simple sample size formula for analysis of covariance in randomized clinical trials. J Clin Epidemiol 2007; 60:1234-8.
20. Nielsen FA, Hansen LK: Modeling of activation data in the BrainMap database: detection of outliers. Hum Brain Mapp. 2002;15:146-56.
21. Schubert R, Ritter P, Wüstenberg T, Preuschhof C, Curio G, Sommer W, Villringer A: Spatial attention related SEP amplitude modulations covary with BOLD signal in S1--a simultaneous EEG--fMRI study. Cereb Cortex. 2008;18:2686-700.
22. Turkeltaub PE, Eden GF, Jones KM, Zeffiro TA: Meta-analysis of the functional neuroanatomy of single-word reading: method and validation. Neuroimage. 2002;16:765-80.
23. Krogias C, Strassburger K, Eyding J, Gold R, Norra C, Juckel G, Saft C, Ninphius D:Depression in patients with Huntington disease correlates with alterations of the brain stem raphe depicted by transcranial sonography.J Psychiatry Neurosci. 2010: 35:100067.
24. Marin RS, Biedrzycki RC, Firinciogullari S: Reliability and validity of the Apathy Evaluation Scale. Psychiatry Res. 1991; 38:143-62.
25. Cummings JL, Mega M, Gray K, Rosenberg-Thompson S, Carusi DA, Gornbein J: The Neuropsychiatric Inventory: comprehensive assessment of psychopathology in dementia. Neurology.1994;44:2308-14.
26. Zigmond AS, Snaith RP: The hospital anxiety and depression scale. Acta Psychiatr Scand. 1983; 67:361-70.
27. Snaith RP, Constantopoulos AA, Jardine MY, McGuffin P. A clinical scale for the self-assessment of irritability. Br J Psychiatry. 1978;132:164-71.
28. Breiter HC, Aharon I, Kahneman D, Dale A, Shizgal P. FMRI of neural responses to expectancy and experience of monetary gains and losses. 2001; Neuron. 30:619-39.
29. Tisdall MD. Prospective Motion Correction of Anatomical Brain Sequences using EPI Navigators, ISMRM Workshop on Current Concepts of Motion Correction for MRI & MRS 2010
