# Supplementary material for: Bupropion for the treatment of apathy in Huntington’s disease: A multicenter, randomised, double-blind, placebo-controlled, prospective crossover trial
Source: PLoS One. 2017 Mar 21;12(3):e0173872. doi: 10.1371/journal.pone.0173872 (PMC5360242; doi:10.1371/journal.pone.0173872)
Supplement: S1 Supplemental methods — (DOCX) [file pone.0173872.s003.docx]

Supplemental Methods

**Gambling Task:** For fMRI, we used an established task known for massive dopaminergic brain response in VS (Lorenz et al., 2014; McCarrey et al., 2012). The task consists in a virtual, physically valid, three cylinder slot machine gamble with a winning probability of about 25%. During fMRI-experiment participants accomplished 80 gambles of about 15 seconds duration each. A button press started the virtual machine. After start, cylinders were accelerated until their final speed. Acceleration rates were fixed exponentially. Thus the final speed of the middle cylinder was twofold and that of the right cylinder fourfold that of the left cylinder. In contrast, the deceleration of all cylinders was identical. Thus, the left cylinder stops two, the middle four and the right eight seconds after participants pressed the stop button. The final cylinder configuration was displayed for one second. Given this temporal characteristic, gain anticipation periods were four seconds. Between games flexible breaks ranging from two to fife seconds duration were inserted. Within this time, a fixation cross was displayed. The whole experiment take about 20 minutes of time. The virtual slot machine was programmed in Presentation® (Ver. 0.71; Neurobehavioral systems; https://www.neurobs.com/) and presented via MRI compatible video goggles with a spatial resolution of 800 x 600 pixels and a vertical refresh rate of 85 Hz (NordicNeuroLab, http://www.advancedmedicalequipment.com). For machine control, we used a fiber optic response button (fORP, Current Design, http://www.curdes.com). In supplemental **S1 Fig.** the slot machine game is shown in detail.

**Computation of literature based probabilistic ROIs:** *A-priori* Regions of Interest (ROIs) for small volume alpha error adjustment were created combining anatomical hypotheses with functional findings as reported in the literature for comparable experimental designs. Firstly, spatial coordinates for the following ROIs were taken from fMRI publications using comparable reward paradigms: ventral striatum (VS), anterior cingulate cortex (ACC), medial prefrontal cortex (MPFC), orbitofrontal cortex (OFC),. Secondly, anatomical ROIs from the Anatomical Automatic Labeling (AAL; http://www.cyceron.fr/web/aal_anatomical_automatic_labeling.html, Tzourio-Mazoyer et al. 2002) were used for all above-mentioned ROIs except the VTA, which was traced manually on the mean anatomical image of the sample by one of the authors (TW). For midline structures (ACC, MPFC), left and right hemispheric coordinates were collapsed to form a bi-hemispheric ROI for each of these structures, whereas all other ROIs were computed separately for the left and right hemisphere. Based on this data set, we create the ROIs in a three-step process (Schubert et al., 2007):


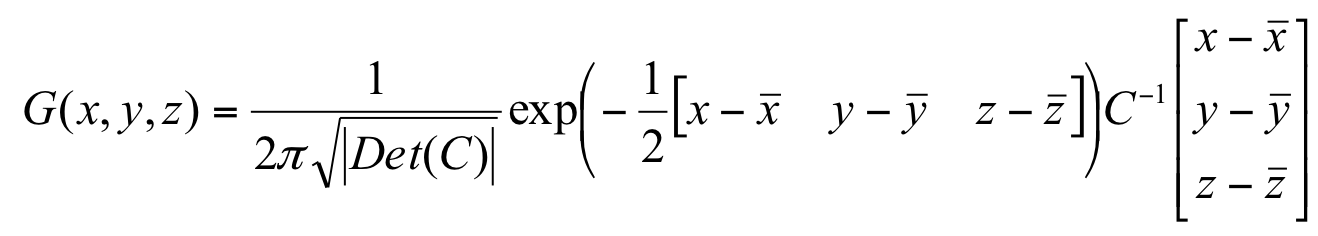
(1) The probability that a voxel at a given position within an anatomical ROI showed neural activity regarding the corresponding literature was estimated by calculating a 3D normal (Gaussian) distribution G(x, y, z) as follows (Turkeltaub et al., 2002):

where C is the covariance matrix for all coordinate triples x, y, z from the underlying literature and x, y, z are the mean values of the x, y, and z coordinates, respectively (Nielsen et al., 2002). (2) The outer limits of the finally used ROI were defined by (a) the outer limits of the anatomical ROI and (b) a threshold of 2 standard deviations of the resulting 3D distribution. (3) Finally a binary mask including all voxels spatially within these boundaries was formed.

Specifically for spatially extended anatomical ROIs (e.g. MPFC, ACC or OFC) containing probably different functional sub regions, this procedure leads to a spatially reduction to design relevant coordinates within these ROIs. Resulting ROIs and their center coordinates as well volume are displayed in Supplementary **Figure S3**. [Note: The script for generating the probabilistic ROIs (written in MatLab by author TW) and the full lists of coordinates used for ROI generation can be obtained from the authors upon request].

**MRI acquisition*:*** 18 out of the 40 enrolled HD took part in MRI-experiments. MRI data were acquired on a 3 Tesla Siemens MAGNETOM Tim Trio MRI system (Siemens, Erlangen, Germany) taken the suggestions of Klöppel and colleagues under account (AR11). First, T1-weighted three-dimensional inversion recovery, turbo flash pulse sequence with isotropic spatial resolution of 1 mm^3^ (image matrix = 256 x 256 x 192, inversion time = 900 ms, repetition time = 1900 ms, echo time = 2.52 ms, flip angle = 9°) was acquired. Secondly, to quantify distortions of the magnetic field a two-dimensional multi-echo gradient-echo B0-field map, with an anisotropic spatial resolution of 3.5 x 3.5 x 4.5 mm^3^ was acquired (image matrix = 64 x 64, number of slices = 30, repetition time = 434 ms, echo times = 5.19 ms & 7.65 ms). Thirdly, 500 T2*-weighted blood oxygenation sensitive (BOLD) hole brain images were acquired by means of a two-dimensional gradient-echo echo-planar imaging (GE-EPI) pulse sequence with the same spatial resolution, slice positioning and slice orientation as the Bo-field map images (repetition time = 2 s, echo time = 30 ms, flip angle = 76°).

**IMAGE PROCESSING - STRUCTURAL MRI:** To account for possible additional image noise due to chorea, for each HD-patient the structural image from 2^nd^ MRI-session was aligned to the structural image of the 1^st^ MRI-session and both images were averaged. Thus, we tried Afterwards, individual anatomical images were classified into gray matter, white matter and cerebrospinal fluid as well as three extra-cerebral tissue classes and transformed into MNI standard space as provided by the International Consortium for Brain Mapping (6 tissue class template; http://www.loni.usc.edu/ICBM) using the diffeomorphic image registration algorithm (DARTEL) developed by Ashburner with spatial resolution of 1.5 x 1.5 x 1.5 mm^3^. To account for local normalization amount, gray matter images were modulated with the nonlinear transformation parameters as computed during normalization procedures. Resulting images contain the volume proportion of probabilistically assigned gray matter tissue for each voxel. These gray matter tissue probability maps (TPMs) were again inspected visually and spatially filtered with an isotropic Gaussian kernel with full-width at a half-maximum of 8 mm. Note that each image of regional GM volume was corrected for individual brain size, since this step is part of the VBM8 toolbox routine. Finally, for display purposes an average anatomical image for the whole MRI-sample was computed.

**IMAGE PROCESSING – FMRI:** Functional images were corrected for acquisition delay, head motions and geometric deformations due to nonlinear distortions of scanners magnetic field (Anderson et al., 2001). Afterward, the structural image was coregistered to the mean artifact corrected functional image computed at the end of the preceding processing step. Forward transformation parameters estimated as described in the VBM-section, were used for the spatial warping of functional images into MNI-space. Finally, preprocessed functional images in native space as well as in MNI-space were spatially filtered with an isotropic Gaussian kernel with 8 mm full width at half maximum and high-pass filtered with a cut-off frequency of 128 seconds. Serial correlations from aliased cardiological and respiratory effects were accounted for using a 1st-order autoregressive model.

**MODELING OF GAIN ANTICIPATION ASSOCIATED BRAIN RESPONSES – FMRI:** By means of the canonical hemodynamic response function as implemented in SPM (HRF, Friston et al. 1995a & b) stimulation locked brain responses were modeled for the following experimental conditions of interest: Gain anticipation (GA) and no gain anticipation (nGA). To reduce further error variance also the gain accomplished and not accomplished gain, the amount of slot machine’s cylinder movements, button presses when starting the machine, button presses when stopping the machine, and six movement parameters were modeled. Finally, a single constant represents the mean over scans. This model was fitted voxel-wise into the fMRI time series using the restricted maximum likelihood (ReML) algorithm as implemented in SPM12. Linear contrast images were computed for the comparison of interest GA – nGA and used for group level analyses.

**MODELING OF GAIN ANTICIPATION ASSOCIATED FUNCTIONAL BRAIN CONNECTIVITY – FMRI:** To investigate how treatment affects connectivity strength between brain regions, we conducted a functional connectivity analysis by means of the Psycho-physiological Interaction (PPI) approach (Friston et al., 1997; Gitelman et al., 2003). For each patient, mean first eigenvariate time series were extracted from voxels within the left and right VS-ROI. The resulting time courses were then convolved with a psychological function P of the time t, which was set to +1 if t was the onset of a gain anticipation period and to -1 if t was the onset of a period without gain anticipation, and 0 in all other cases. The re-convolution of the resulting function with the HRF yielded two vectors X that formed the primary regressors of interest in the design matrix of a new single subject GLM. The psychological variable P was also convolved with the HRF to form an additional regressor. The third explanatory variable was the original BOLD eigenvariate time series as extracted from the seed region. The remaining conditions, the six rigid-body movement parameters determined from head motion correction and a constant representing the mean over scans was included in the design matrix as covariates of no interest. Model estimation was performed as described above, separately for the two seed regions. Voxel-wise linear contrast images for the comparison GA – nGA were computed and used for group level analyses.

**VOXEL-WISE ANALYSES OF TREATMENT EFFECTS:** For all MRI-outcomes (local gray matter volume/TPM, linear contrast images GA – nGA for brain response and functional connectivity), we conducted separate voxel-wise Analyses of covariance (ANCOVAs) for repeated measures comprising the experimental factors TIME (repetition factor) and TREATMENT (between subjects factor) as well as age and gender (brain structure only) or chorea severity (brain response and connectivity only) as a covariate of no interest. Voxels with and interactions between TIME and TREATMENT were identified via F-testing (p < .05 uncorrected, no cluster threshold). Alpha error probabilities were adjusted for ROI volume by means of small volume correction approach as implemented in SPM12. Uncorrected as well as family-wise error (FEW) corrected alpha error probabilities are listed in S1 Table – S3 Table. For ROIs showing at least a FEW-correctable trend toward an interaction, *post-hoc* t-tests were conducted. Only voxels passing a threshold of p < .05 (family-wise error corrected for multiple comparisons) in F- or t-testing were considered for discussion.

A detailed description of MR-image processing and analyses can be found in S2 Fig**.**

Supplemental References

Andersson JLR, Hutton C, Ashburner J, Turner R, Friston K (2001) Modelling geometric deformations in EPI time series. NeuroImage 13:903-919

Friston, K.J., Frith, C.D., Turner, R., and Frackowiak, R.S. (1995a). Characterizing evoked hemodynamics with fMRI. NeuroImage *2*, 157–165.

Friston, K.J., Holmes, A.P., Poline, J.B., Grasby, P.J., Williams, S.C., Frackowiak, R.S., and Turner, R. (1995b). Analysis of fMRI time-series revisited. NeuroImage *2*, 45–53.

Friston, K.J., Buechel, C., Fink, G.R., Morris, J., Rolls, E., and Dolan, R.J. (1997). Psychophysiological and modulatory interactions in neuroimaging. NeuroImage *6*, 218–229.

Gitelman, D.R., Penny, W.D., Ashburner, J., and Friston, K.J. (2003). Modeling regional and psychophysiologic interactions in fMRI: the importance of hemodynamic deconvolution. NeuroImage *19*, 200–207.

Lorenz RC, Gleich T, Beck A, Pöhland L, Raufelder D, Sommer W, Rapp MA, Kühn S, Gallinat J. Reward anticipation in the adolescent and aging brain. Hum Brain Mapp. 2014 Oct;35(10):5153-65.

McCarrey AC, Henry JD, von Hippel W, Weidemann G, Sachdev PS, Wohl MJ, Williams M. Age differences in neural activity during slot machine gambling: an fMRI study. PLoS One. 2012;7(11):e49787.

**ROI – Methods**

Schubert,R. et al. Spatial Attention Related SEP Amplitude Modulations Covary with BOLD Signal in S1-A Simultaneous EEG-fMRI Study. Cerebral Cortex 18, 2686-2700 (2008)

Turkeltaub,P.E., Eden,G.F., Jones,K.M. & Zeffiro,T.A. Meta-analysis of the functional neuroanatomy of single-word reading: Method and validation. Neuroimage 16:765-780 (2002).

Nielsen,F.A. & Hansen,L.K. Modeling of activation data in the BrainMap (TM) database: Detection of outliers. Human Brain Mapping 15:146-156 (2002).

Tzourio-Mazoyer N, Landeau B, Papathanassiou D, Crivello F, Etard O, Delcroix N, Mazoyer B and Joliot M. Automated Anatomical Labeling of activations in SPM using a Macroscopic Anatomical Parcellation of the MNI MRI single-subject brain". NeuroImage 15:273–289 (2002).

**ROI – Literature**

**Mesolimbic regions**

**Left Ventral striatum (VS)**

Bjork JM, Knutson B, Fong GW, Caggiano DM, Bennett SM, et al. (2004) Incentive-elicited brain activation in adolescents: similarities and differences from young adults. J Neurosci 24: 1793-1802.

Bjork JM, Smith AR, Chen G, Hommer DW (2010) Adolescents, adults and rewards: comparing motivational neurocircuitry recruitment using fMRI. PLoS One 5: e11440.

Camara E, Rodriguez-Fornells A, Munte TF (2008) Functional connectivity of reward processing in the brain. Front Hum Neurosci 2: 19.

Cooper JC, Hollon NG, Wimmer GE, Knutson B (2009) Available alternative incentives modulate anticipatory nucleus accumbens activation. Soc Cogn Affect Neurosci 4: 409-416.

Dichter GS, Felder JN, Green SR, Rittenberg AM, Sasson NJ, et al. (2010) Reward circuitry function in autism spectrum disorders. Soc Cogn Affect Neurosci.

Juckel G, Schlagenhauf F, Koslowski M, Filonov D, Wustenberg T, et al. (2006) Dysfunction of ventral striatal reward prediction in schizophrenic patients treated with typical, not atypical, neuroleptics. Psychopharmacology (Berl) 187: 222-228.

Juckel G, Schlagenhauf F, Koslowski M, Wustenberg T, Villringer A, et al. (2006) Dysfunction of ventral striatal reward prediction in schizophrenia. Neuroimage 29: 409-416.

Knutson B, Fong GW, Bennett SM, Adams CM, Hommer D (2003) A region of mesial prefrontal cortex tracks monetarily rewarding outcomes: characterization with rapid event-related fMRI. Neuroimage 18: 263-272.

Knutson B, Bjork JM, Fong GW, Hommer D, Mattay VS, et al. (2004) Amphetamine modulates human incentive processing. Neuron 43: 261-269.

Knutson B, Taylor J, Kaufman M, Peterson R, Glover G (2005) Distributed neural representation of expected value. J Neurosci 25: 4806-4812.

Knutson B, Rick S, Wimmer GE, Prelec D, Loewenstein G (2007) Neural predictors of purchases. Neuron 53: 147-156.

Knutson B, Bhanji JP, Cooney RE, Atlas LY, Gotlib IH (2008) Neural responses to monetary incentives in major depression. Biol Psychiatry 63: 686-692.

Muhlberger A, Wieser MJ, Gerdes AB, Frey MC, Weyers P, et al. (2011) Stop looking angry and smile, please: start and stop of the very same facial expression differentially activate threat- and reward-related brain networks. Soc Cogn Affect Neurosci 6: 321-329.

Ossewaarde L, Qin S, Van Marle HJ, van Wingen GA, Fernandez G, et al. (2011) Stress-induced reduction in reward-related prefrontal cortex function. Neuroimage 55: 345-352.

Rademacher L, Krach S, Kohls G, Irmak A, Grunder G, et al. (2010) Dissociation of neural networks for anticipation and consumption of monetary and social rewards. Neuroimage 49: 3276-3285.

Stark R, Bauer E, Merz CJ, Zimmermann M, Reuter M, et al. (2011) ADHD related behaviors are associated with brain activation in the reward system. Neuropsychologia 49: 426-434.

Stoppel CM, Boehler CN, Strumpf H, Heinze HJ, Hopf JM, et al. (2011) Neural processing of reward magnitude under varying attentional demands. Brain Res 1383: 218-229.

**Right Ventral striatum (VS)**

Andrews MM, Meda SA, Thomas AD, Potenza MN, Krystal JH, et al. (2011) Individuals family history positive for alcoholism show functional magnetic resonance imaging differences in reward sensitivity that are related to impulsivity factors. Biol Psychiatry 69: 675-683.

Bjork JM, Knutson B, Fong GW, Caggiano DM, Bennett SM, et al. (2004) Incentive-elicited brain activation in adolescents: similarities and differences from young adults. J Neurosci 24: 1793-1802.

Bjork JM, Smith AR, Chen G, Hommer DW (2010) Adolescents, adults and rewards: comparing motivational neurocircuitry recruitment using fMRI. PLoS One 5: e11440.

Camara E, Rodriguez-Fornells A, Munte TF (2008) Functional connectivity of reward processing in the brain. Front Hum Neurosci 2: 19.

Cooper JC, Hollon NG, Wimmer GE, Knutson B (2009) Available alternative incentives modulate anticipatory nucleus accumbens activation. Soc Cogn Affect Neurosci 4: 409-416.

Juckel G, Schlagenhauf F, Koslowski M, Filonov D, Wustenberg T, et al. (2006) Dysfunction of ventral striatal reward prediction in schizophrenic patients treated with typical, not atypical, neuroleptics. Psychopharmacology (Berl) 187: 222-228.

Juckel G, Schlagenhauf F, Koslowski M, Wustenberg T, Villringer A, et al. (2006) Dysfunction of ventral striatal reward prediction in schizophrenia. Neuroimage 29: 409-416.

Knutson B, Adams CM, Fong GW, Hommer D (2001) Anticipation of increasing monetary reward selectively recruits nucleus accumbens. J Neurosci 21: RC159.

Knutson B, Fong GW, Bennett SM, Adams CM, Hommer D (2003) A region of mesial prefrontal cortex tracks monetarily rewarding outcomes: characterization with rapid event-related fMRI. Neuroimage 18: 263-272.

Knutson B, Bjork JM, Fong GW, Hommer D, Mattay VS, et al. (2004) Amphetamine modulates human incentive processing. Neuron 43: 261-269.

Knutson B, Taylor J, Kaufman M, Peterson R, Glover G (2005) Distributed neural representation of expected value. J Neurosci 25: 4806-4812.

Knutson B, Rick S, Wimmer GE, Prelec D, Loewenstein G (2007) Neural predictors of purchases. Neuron 53: 147-156.

Knutson B, Bhanji JP, Cooney RE, Atlas LY, Gotlib IH (2008) Neural responses to monetary incentives in major depression. Biol Psychiatry 63: 686-692.

Muhlberger A, Wieser MJ, Gerdes AB, Frey MC, Weyers P, et al. (2011) Stop looking angry and smile, please: start and stop of the very same facial expression differentially activate threat- and reward-related brain networks. Soc Cogn Affect Neurosci 6: 321-329.

Rademacher L, Krach S, Kohls G, Irmak A, Grunder G, et al. (2010) Dissociation of neural networks for anticipation and consumption of monetary and social rewards. Neuroimage 49: 3276-3285.

Stark R, Bauer E, Merz CJ, Zimmermann M, Reuter M, et al. (2011) ADHD related behaviors are associated with brain activation in the reward system. Neuropsychologia 49: 426-434.

Stoppel CM, Boehler CN, Strumpf H, Heinze HJ, Hopf JM, et al. (2011) Neural processing of reward magnitude under varying attentional demands. Brain Res 1383: 218-229.

Strohle A, Stoy M, Wrase J, Schwarzer S, Schlagenhauf F, et al. (2008) Reward anticipation and outcomes in adult males with attention-deficit/hyperactivity disorder. Neuroimage 39: 966-972.

**Left and right medial prefrontal cortex (MPFC)**

Bjork JM, Knutson B, Fong GW, Caggiano DM, Bennett SM, et al. (2004) Incentive-elicited brain activation in adolescents: similarities and differences from young adults. J Neurosci 24: 1793-1802.

Bjork JM, Smith AR, Chen G, Hommer DW (2010) Adolescents, adults and rewards: comparing motivational neurocircuitry recruitment using fMRI. PLoS One 5: e11440.

Bolla KI, Eldreth DA, London ED, Kiehl KA, Mouratidis M, et al. (2003) Orbitofrontal cortex dysfunction in abstinent cocaine abusers performing a decision-making task. Neuroimage 19: 1085-1094.

Dillon DG, Bogdan R, Fagerness J, Holmes AJ, Perlis RH, et al. (2010) Variation in TREK1 gene linked to depression-resistant phenotype is associated with potentiated neural responses to rewards in humans. Hum Brain Mapp 31: 210-221.

Goldin PR, McRae K, Ramel W, Gross JJ (2008) The neural bases of emotion regulation: reappraisal and suppression of negative emotion. Biol Psychiatry 63: 577-586.

Knutson B, Adams CM, Fong GW, Hommer D (2001) Anticipation of increasing monetary reward selectively recruits nucleus accumbens. J Neurosci 21: RC159.

Knutson B, Fong GW, Bennett SM, Adams CM, Hommer D (2003) A region of mesial prefrontal cortex tracks monetarily rewarding outcomes: characterization with rapid event-related fMRI. Neuroimage 18: 263-272.

Knutson B, Bjork JM, Fong GW, Hommer D, Mattay VS, et al. (2004) Amphetamine modulates human incentive processing. Neuron 43: 261-269.

Knutson B, Taylor J, Kaufman M, Peterson R, Glover G (2005) Distributed neural representation of expected value. J Neurosci 25: 4806-4812.

Ossewaarde L, Qin S, Van Marle HJ, van Wingen GA, Fernandez G, et al. (2011) Stress-induced reduction in reward-related prefrontal cortex function. Neuroimage 55: 345-352.

Padmala S, Pessoa L (2011) Reward reduces conflict by enhancing attentional control and biasing visual cortical processing. J Cogn Neurosci 23: 3419-3432.

Stoppel CM, Boehler CN, Strumpf H, Heinze HJ, Hopf JM, et al. (2011) Neural processing of reward magnitude under varying attentional demands. Brain Res 1383: 218-229.

Strohle A, Stoy M, Wrase J, Schwarzer S, Schlagenhauf F, et al. (2008) Reward anticipation and outcomes in adult males with attention-deficit/hyperactivity disorder. Neuroimage 39: 966-972.

Viard A, Doeller CF, Hartley T, Bird CM, Burgess N (2011) Anterior hippocampus and goal-directed spatial decision making. J Neurosci 31: 4613-4621.

**Left orbitofrontal cortex (OFC)**

Camara E, Rodriguez-Fornells A, Munte TF (2008) Functional connectivity of reward processing in the brain. Front Hum Neurosci 2: 19.

Dichter GS, Felder JN, Green SR, Rittenberg AM, Sasson NJ, et al. (2010) Reward circuitry function in autism spectrum disorders. Soc Cogn Affect Neurosci.

Franklin T, Wang Z, Suh JJ, Hazan R, Cruz J, et al. (2011) Effects of varenicline on smoking cue-triggered neural and craving responses. Arch Gen Psychiatry 68: 516-526.

Knutson B, Taylor J, Kaufman M, Peterson R, Glover G (2005) Distributed neural representation of expected value. J Neurosci 25: 4806-4812.

O'Doherty J, Kringelbach ML, Rolls ET, Hornak J, Andrews C (2001) Abstract reward and punishment representations in the human orbitofrontal cortex. Nat Neurosci 4: 95-102.

Sescousse G, Redoute J, Dreher JC (2010) The architecture of reward value coding in the human orbitofrontal cortex. J Neurosci 30: 13095-13104.

Simon JJ, Walther S, Fiebach CJ, Friederich HC, Stippich C, et al. (2010) Neural reward processing is modulated by approach- and avoidance-related personality traits. Neuroimage 49: 1868-1874.

Stark R, Bauer E, Merz CJ, Zimmermann M, Reuter M, et al. (2011) ADHD related behaviors are associated with brain activation in the reward system. Neuropsychologia 49: 426-434.

Stoppel CM, Boehler CN, Strumpf H, Heinze HJ, Hopf JM, et al. (2011) Neural processing of reward magnitude under varying attentional demands. Brain Res 1383: 218-229.

Wrase J, Kahnt T, Schlagenhauf F, Beck A, Cohen MX, et al. (2007) Different neural systems adjust motor behavior in response to reward and punishment. Neuroimage 36: 1253-1262.

**Right orbitofrontal cortex (OFC)**

Acevedo BP, Aron A, Fisher HE, Brown LL (2011) Neural correlates of long-term intense romantic love. Soc Cogn Affect Neurosci.

Andrews MM, Meda SA, Thomas AD, Potenza MN, Krystal JH, et al. (2011) Individuals family history positive for alcoholism show functional magnetic resonance imaging differences in reward sensitivity that are related to impulsivity factors. Biol Psychiatry 69: 675-683.

Camara E, Rodriguez-Fornells A, Munte TF (2008) Functional connectivity of reward processing in the brain. Front Hum Neurosci 2: 19.

Dichter GS, Felder JN, Green SR, Rittenberg AM, Sasson NJ, et al. (2010) Reward circuitry function in autism spectrum disorders. Soc Cogn Affect Neurosci.

Knutson B, Taylor J, Kaufman M, Peterson R, Glover G (2005) Distributed neural representation of expected value. J Neurosci 25: 4806-4812.

Simon JJ, Walther S, Fiebach CJ, Friederich HC, Stippich C, et al. (2010) Neural reward processing is modulated by approach- and avoidance-related personality traits. Neuroimage 49: 1868-1874.

Stark R, Bauer E, Merz CJ, Zimmermann M, Reuter M, et al. (2011) ADHD related behaviors are associated with brain activation in the reward system. Neuropsychologia 49: 426-434.

Stoppel CM, Boehler CN, Strumpf H, Heinze HJ, Hopf JM, et al. (2011) Neural processing of reward magnitude under varying attentional demands. Brain Res 1383: 218-229.

Strohle A, Stoy M, Wrase J, Schwarzer S, Schlagenhauf F, et al. (2008) Reward anticipation and outcomes in adult males with attention-deficit/hyperactivity disorder. Neuroimage 39: 966-972.

Wrase J, Kahnt T, Schlagenhauf F, Beck A, Cohen MX, et al. (2007) Different neural systems adjust motor behavior in response to reward and punishment. Neuroimage 36: 1253-1262.
