# Supplementary figures and images for: Bupropion for the treatment of apathy in Huntington’s disease: A multicenter, randomised, double-blind, placebo-controlled, prospective crossover trial
Source: PLoS One. 2017 Mar 21;12(3):e0173872. doi: 10.1371/journal.pone.0173872 (PMC5360242; doi:10.1371/journal.pone.0173872)

S1 Fig.: Study design

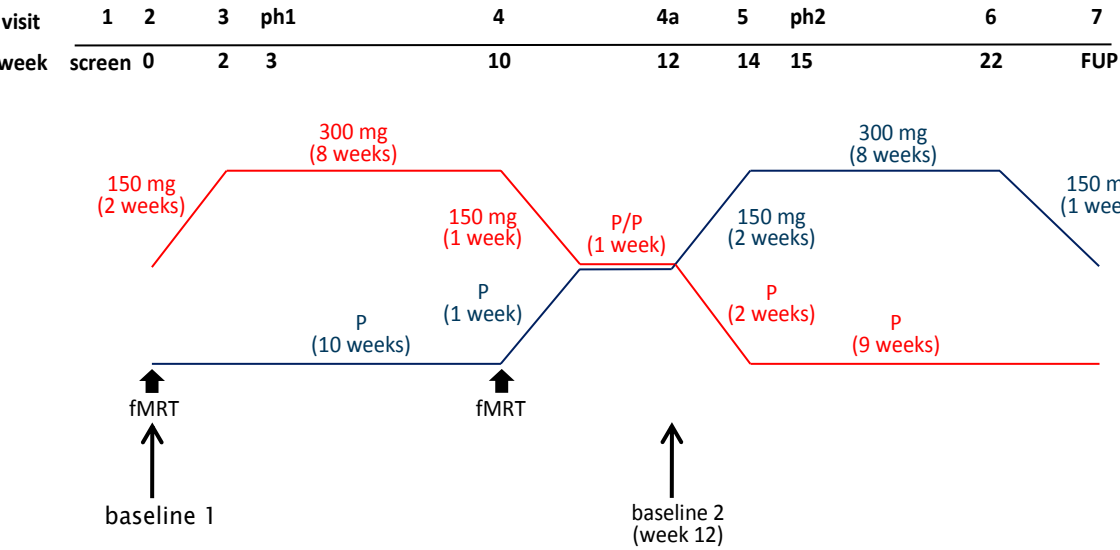

Supplement: S1 Fig — (PDF) [file pone.0173872.s004.pdf]

S2 Fig.: Experimental set up: Slot machine paradigm.

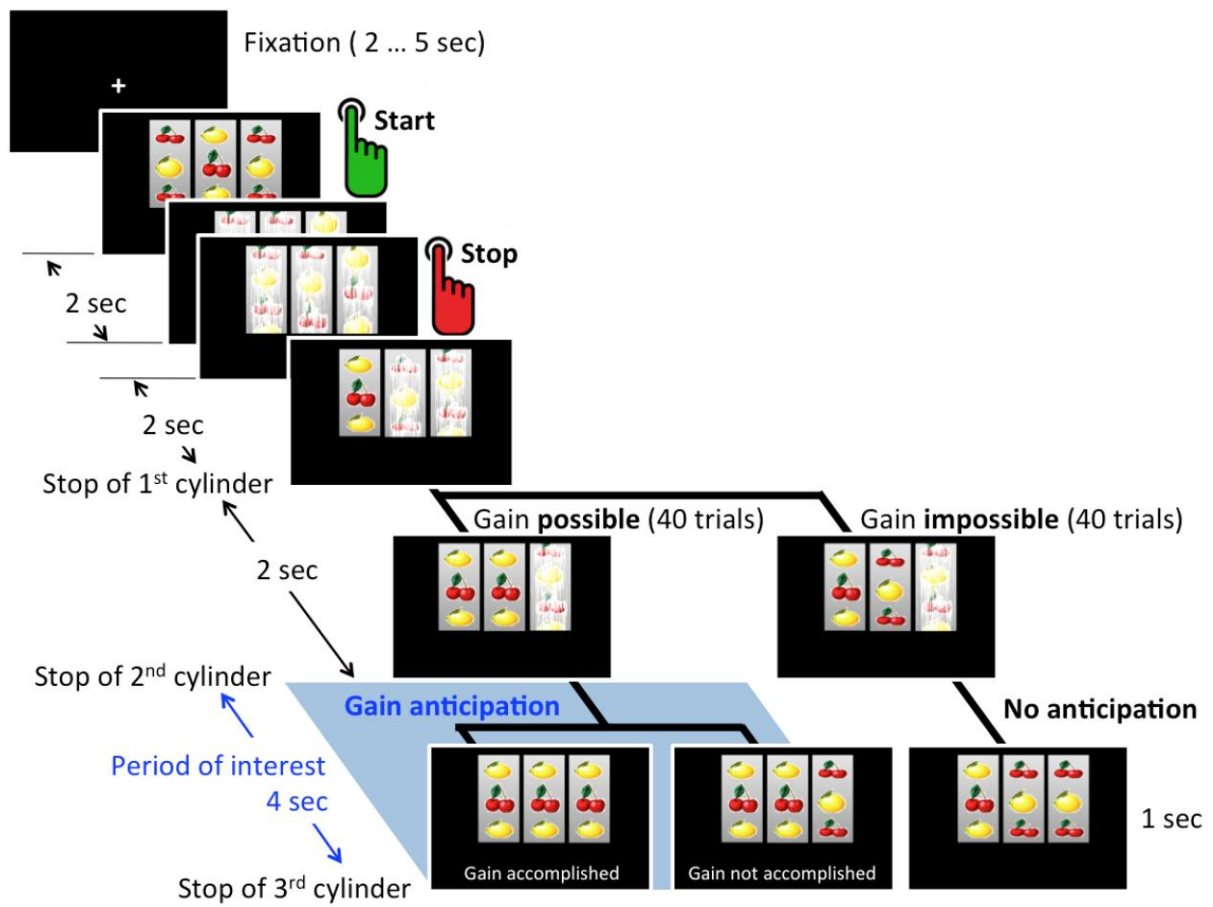

Supplement: S2 Fig — The period of interest (gain anticipation) is displayed in blue. (PDF) [file pone.0173872.s005.pdf]

S3 Fig.: MR-image analysis pipeline.

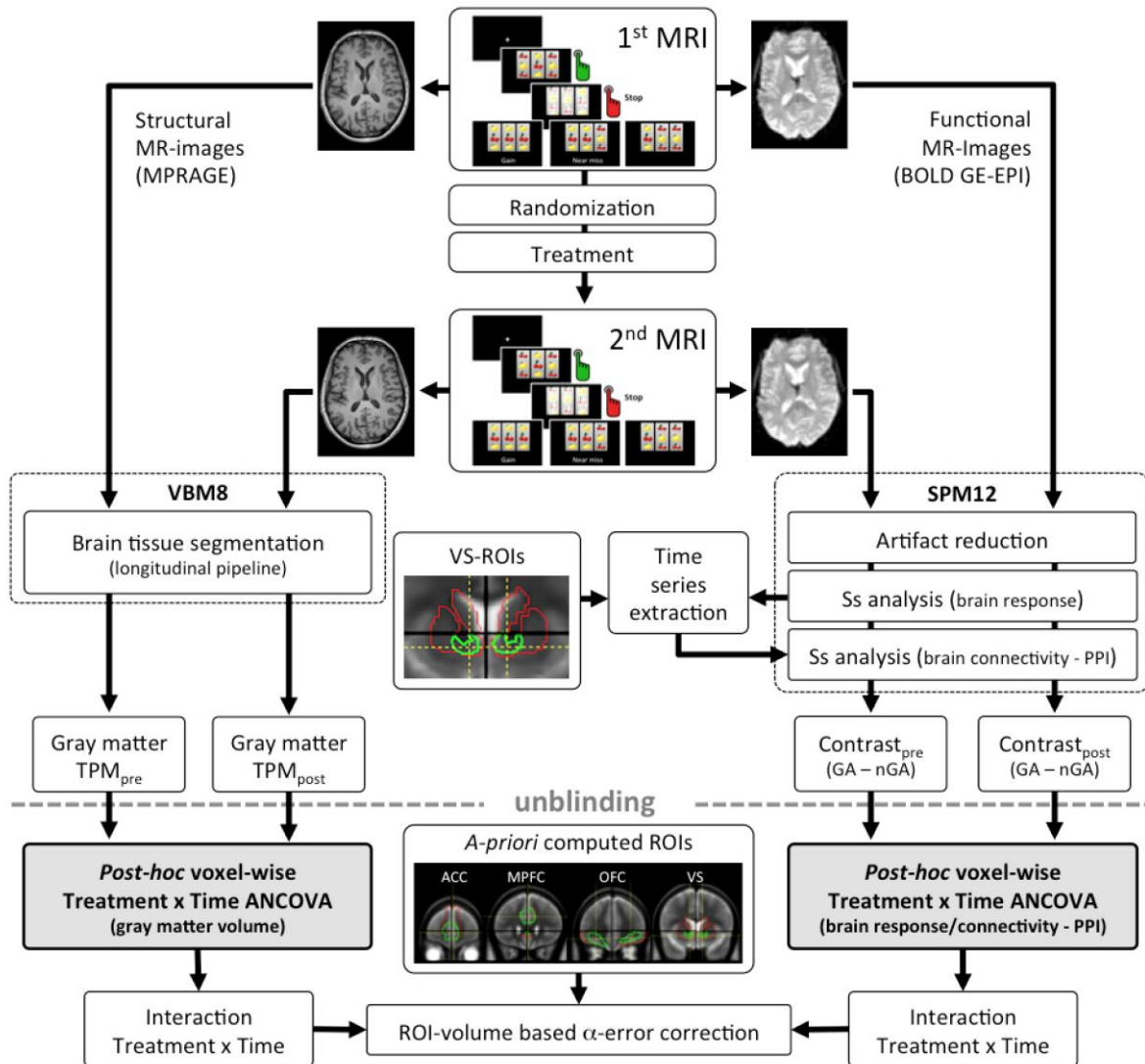

Supplement: S3 Fig — Abbreviations: MRI—Magnetic resonance imaging; Ss—single subject; MNI—spatial reference space as defined by the brain template of the Montreal Neurological Institute; ANCOVA—Analysis of covariance, MPRAGE—Magnetization prepared rapid-acquisition gradient-echo image; BOLD—Blood oxygenation dependent; GE-EPI—Gradient-echo echo-planar image, VBM—Voxel-based morphometry, SPM—Statistical parametrical mapping, TPM—Tissue probability map, GA—Gain anticipation, nGA—no Gain anticipation, ROI—Region of Interest, ANCOVA—Analysis of Covariance, VS—Ventral striatum, ACC—Anterior cingulate cortex, MPFC—Medial prefrontal cortex, OFC—Orbitofrontal cortex. (PDF) [file pone.0173872.s006.pdf]

S4 Fig.: *A-priori* computed literature-based, probabilistic regions of interest (ROIs).

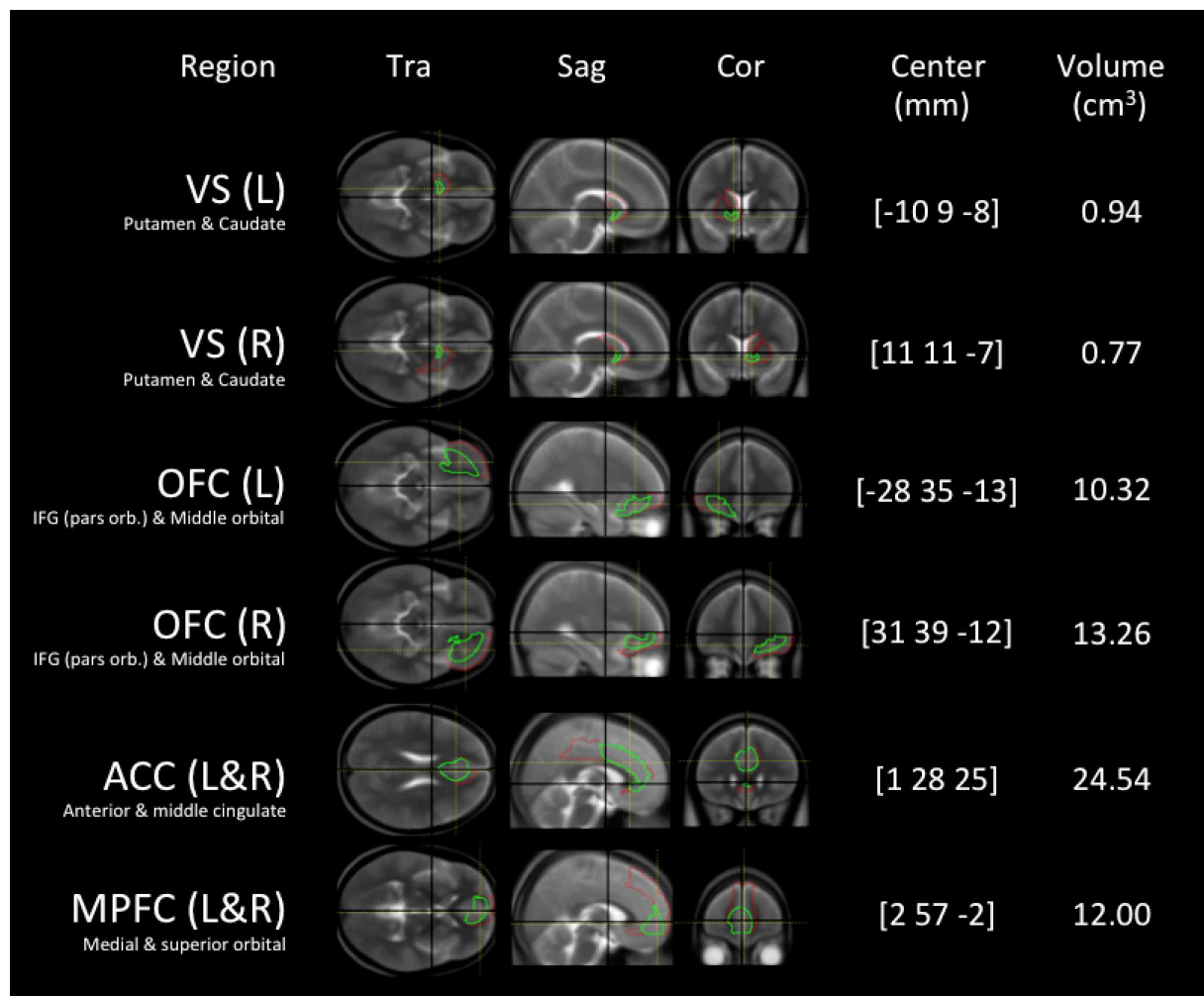

Supplement: S4 Fig — Displayed are tri-orthogonal cuts through the ROI center. The outer borders (2SD) of the probabilistic ROIs are displayed in green; the outer borders of the anatomical structures/constraints taken from the AAL-atlas are displayed in red. ROI name and the used anatomical constraints are listed left hand, MNI-center coordinates and volume of the ROIs are listed in the right hand part. Abbreviations: AAL—Automatically anatomical labeling, MNI—Reference space according to the brain template provided by the Montreal Neurological Institute, Tra—Transversal, Sag—Sagittal, Cor—Coronal, VS—Ventral striatum, ACC—Anterior cingulate cortex, MPFC—Medial prefrontal cortex, OFC—Orbitofrontal cortex, L—Left, R—Right. (PDF) [file pone.0173872.s007.pdf]
