# Supplementary material for: Bupropion for the treatment of apathy in Huntington’s disease: A multicenter, randomised, double-blind, placebo-controlled, prospective crossover trial
Source: PLoS One. 2017 Mar 21;12(3):e0173872. doi: 10.1371/journal.pone.0173872 (PMC5360242; doi:10.1371/journal.pone.0173872)
Supplement: S1 Table — Interaction between TREATMENT and TIME as revealed by voxel-wise ANCOVA (p < .05 uncorrected, covariates age & sex). Alpha-errors adjusted post-hoc for ROI-volume. Post-hoc comparison V(pre<post) > P(pre<post). Abbreviations: FEW—Family-wise error, V—Verum, P—Placebo, R—Right, L—Left. (DOCX) [file pone.0173872.s008.docx]

**S1 Table: Treatment associated effects on brain structure (local gray matter volume).**

| **Region of interest** | **Cluster size (mm^3^)** | **F(1,11)**  **peak** | **p** | **p_FWE_** | **Post-hoc**  **T(p_FWE_)** |
| --- | --- | --- | --- | --- | --- |
|  | | | | | |
|  | | | | | |
| Ventral Striatum (L) | No suprathreshold (p < .05 uncorr.) voxels in ROI | | | | |
| Ventral Striatum (R) | No suprathreshold (p < .05 uncorr.) voxels in ROI | | | | |
| Anterior Cingulate Cortex (L/R) | 1539 | 35.23 | <.001 | .148 |  |
| Medial Prefrontal Cortex (L/R) | 921 | 27.75 | <.001 | .180 |  |
| Orbitofrontal Cortex (L) | 243 | 13.09 | .004 | .702 |  |
| Orbitofrontal Cortex (R) | **2123** | **35.43** | **<.001** | **.088** | **-5.95 (.045)** |
|  | | | | | |
