# Supplementary material for: Bupropion for the treatment of apathy in Huntington’s disease: A multicenter, randomised, double-blind, placebo-controlled, prospective crossover trial
Source: PLoS One. 2017 Mar 21;12(3):e0173872. doi: 10.1371/journal.pone.0173872 (PMC5360242; doi:10.1371/journal.pone.0173872)
Supplement: S2 Table — Interaction between TREATMENT and TIME as revealed by voxel-wise ANCOVA (p < .05 uncorrected, covariates chorea severity). Alpha-errors adjusted post-hoc for ROI-volume. Post-hoc comparison V(pre<post) > P(pre<post). Abbreviations: FEW—Family-wise error, V—Verum, P—Placebo, R—Right, L—Left. (DOCX) [file pone.0173872.s009.docx]

**Supplemental Table S2: Treatment associated effects on brain response (GA – nGA).**

| **Region of interest** | **Cluster size (mm^3^)** | **F(1,11)**  **peak** | **p** | **p_FWE_** | ***Post-hoc***  **T(p_FWE_)** |
| --- | --- | --- | --- | --- | --- |
|  | | | | | |
|  | | | | | |
| Ventral Striatum (L) | No suprathreshold (p < .05 uncorr.) voxels in ROI | | | | |
| Ventral Striatum (R) | No suprathreshold (p < .05 uncorr.) voxels in ROI | | | | |
| Anterior Cingulate Cortex (L/R) | 25 | 5.44 | .039 | .826 |  |
| Medial Prefrontal Cortex (L/R) | 282 | 11.63 | .006 | .504 |  |
| Orbitofrontal Cortex (L) | 25 | 5.38 | .040 | .862 |  |
| Orbitofrontal Cortex (R) | 12 | 9.66 | .010 | .609 |  |
|  | | | | | |
