# Supplementary material for: Bupropion for the treatment of apathy in Huntington’s disease: A multicenter, randomised, double-blind, placebo-controlled, prospective crossover trial
Source: PLoS One. 2017 Mar 21;12(3):e0173872. doi: 10.1371/journal.pone.0173872 (PMC5360242; doi:10.1371/journal.pone.0173872)
Supplement: S3 Table — Interaction between TREATMENT and TIME as revealed by voxel-wise ANCOVA (p < .05 uncorrected, covariates chorea severity) for left VS (upper part) and right VS (lower part) separately. Alpha-errors adjusted post-hoc for ROI-volume. Post-hoc comparison V(pre< post) > P(pre<post). Abbreviations: PPI—Psycho-physiological interaction, VS—Ventral striatum, FEW—Family-wise error, V—Verum, P—Placebo, R—Right, L—Left. (DOCX) [file pone.0173872.s010.docx]

**Supplemental Table S3: Treatment associated effects on functional brain connectivity (PPI: GA – nGA).**

| **Region of interest** | **Cluster size (mm^3^)** | **F(1,11)**  **peak** | **p** | **p_FWE_** | ***Post-hoc***  **T(p_FWE_)** |
| --- | --- | --- | --- | --- | --- |
|  | | | | | |
|  | | | | | |
| **Ventral Striatum (L)** | **Seed region** | | | | |
| Ventral Striatum (R) | **159** | **14.39** | **.003** | **.068** | **3.79(.034)** |
| Anterior Cingulate Cortex (L/R) | 331 | 14.44 | .003 | .547 |  |
| Medial Prefrontal Cortex (L/R) | 110 | 9.32 | .011 | .716 |  |
| Orbitofrontal Cortex (L) | 331 | 15.14 | .003 | .318 |  |
| Orbitofrontal Cortex (R) | 306 | 10.10 | .009 | .641 |  |
|  |  |  |  |  |  |
|  |  |  |  |  |  |
| Ventral Striatum (L) | No suprathreshold (p < .05 uncorr.) voxels in ROI | | | | |
| **Ventral Striatum (R)** | **Seed region** | | | | |
| Anterior Cingulate Cortex (L/R) | 74 | 8.29 | .015 | .869 |  |
| Medial Prefrontal Cortex (L/R) | No suprathreshold (p < .05 uncorr.) voxels in ROI | | | | |
| Orbitofrontal Cortex (L) | 25 | 6.34 | .028 | .822 |  |
| Orbitofrontal Cortex (R) | No suprathreshold (p < .05 uncorr.) voxels in ROI | | | | |
|  | | | | | |
